# Fatty Acid Binding Protein 5 Mediates Astrocytic Pyroptosis and Neuroinflammation in Epilepsy via cGAS/STING Pathway

**DOI:** 10.1002/advs.76377

**Published:** 2026-07-06

**Authors:** Chen Chen, Yao Zhao, Yangye Lian, Yifei Hou, Lifen Gong, Tingting Wu, Yulin Yang, Jiaqi Zhu, Xin Wang, Kaihua Zhang, Jing Ding

**Affiliations:** ^1^ Department of Neurology Zhongshan Hospital Fudan University Shanghai China; ^2^ Institute For Translational Brain Research Center for Clinical Neuro‐AI State Key Laboratory of Brain Function and Disorders MOE Frontiers Center For Brain Science Fudan University Shanghai China; ^3^ Department of Pediatrics The First Affiliated Hospital Zhejiang University School of Medicine Hangzhou China

**Keywords:** astrocytes, cGAS‐STING pathway, epilepsy, fatty acid binding protein 5, pyroptosis

## Abstract

Pyroptosis is an inflammatory type of programmed cell death that may contribute to epilepsy initiation and progression through neuroinflammation. Fatty acid binding protein 5 (FABP5), a lipid chaperone, has been implicated in chronic inflammation. However, whether FABP5 regulates pyroptosis and its pathological role in epilepsy remains uncharacterized. Here, FABP5 was upregulated in astrocytes from temporal lobe epilepsy (TLE) patients, epileptic mice, and primary cells. Deletion of astrocytic *Fabp5* significantly attenuated pyroptosis, neuronal loss, and seizure activity in epilepsy. Furthermore, cyclic GMP‐AMP synthase (cGAS)‐stimulator of interferon genes (STING) pathway was identified as the downstream signaling of FABP5 by RNA sequencing analysis. Mechanistically, *Fabp5* knockdown reduced lipid overload, alleviated mitochondrial dysfunction, and suppressed cGAS‐STING activation. Pharmacological inhibition of mitochondrial fatty acid import recapitulated these protective effects. In contrast, *Sting* overexpression abolished the reduced pyroptosis level by *Fabp5* knockdown, whereas STING inhibition using C‐176 attenuated pyroptosis and seizure activity. Collectively, these findings revealed the regulatory role of FABP5‐cGAS‐STING‐pyroptosis axis in the progression of epilepsy and highlighted the promising potential of astrocytic FABP5 as a therapeutic target for epilepsy.

## Graphical abstract

Astrocytic FABP5 promotes mitochondrial stress, cGAS‐STING pathway activation, pyroptosis, and neuroinflammation in epilepsy, contributing to seizure pathology. Genetic targeting of FABP5 or pharmacological inhibition of STING alleviates epileptic phenotypes, highlighting a potential therapeutic strategy for epilepsy.

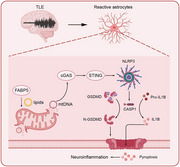

## 1 Introduction

Epilepsy is one of the most common neurological disorders characterized by spontaneous recurrent seizures (SRSs), affecting more than 70 million individuals worldwide [1]. Focal epilepsy accounts for 60% of these cases, among which temporal lobe epilepsy (TLE) originating from the hippocampus or amygdala following an initial brain insult is the most common type [2]. Despite the availability of over 20 antiseizure drugs for treatment, a total of 25% to 50% of patients with epilepsy have seizures refractory to pharmacotherapy [3]. Current antiseizure medications mainly suppress seizure symptoms but do not alter long‐term prognosis due to the complicated pathophysiology in epileptogenesis [4, 5]. Therefore, exploration of medications with new cellular and molecular targets and mechanisms is an urgent clinical need.

Brain inflammation critically contributes to epilepsy initiation and progression. Uncontrolled activation of inflammatory cells involving resident glial cells, and increased levels of chemokines and cytokines in the insulted neuronal tissue are critical factors in the onset and development of epilepsy [6]. Pyroptosis is an inflammatory type of programmed cell death usually triggered through caspase‐1 (CASP1)‐dependent pathway, where the inflammasome is activated and leads to the cleavage of caspase‐1. The activated caspase‐1 is responsible for the production of N‐terminus of gasdermin D (GSDMD‐NT), the final executor of pyroptosis [7, 8]. Immoderate cytokines (e.g., IL‐1β, IL‐18) released during pyroptosis increase neuronal excitability and perpetuate inflammatory responses in epilepsy [9, 10]. Accumulating evidence has shown the elevated protein levels of inflammasome (e.g., NLRP1, NLRP3), CASP1, and GSDMD‐NT in the hippocampus from TLE patients and animal models [11, 12, 13, 14]. Pharmacological inhibition or knockout of inflammasome decreases epilepsy susceptibility, alleviates neuronal damage, glial cell activation, and neurobehavioral impairment [15, 16, 17]. Our previous study has also demonstrated that administration of dimethyl fumarate—a GSDMD‐NT inhibitor, attenuates the reactive activation of astrocytes and SRSs after status epilepticus [18]. Although accumulating evidence has shown an association between pyroptosis and epilepsy, further study is warranted to reveal the molecular mechanism underlying pyroptosis in the context of epilepsy.

Fatty acid‐binding proteins (FABPs) are a family of low molecular weight intracellular proteins and consist of 10 members in mammals (FABP1–9 and FABP12). Among them, FABP3, FABP5, and FABP7 are expressed in the brain [19]. FABPs are traditionally considered as intracellular chaperones for fatty acids and function to facilitate fatty acid trafficking and metabolism and to coordinate lipid responses inside cells [20]. Further investigation has demonstrated their participation, especially FABP4 and FABP5, also in the fields of chronic inflammation, both via lipid metabolism and via regulation of signaling within their cells of expression [21, 22]. FABP5 has been found to upregulate NF‐*k*B signaling in mesothelioma [23] and to promote IL‐1β signaling in psoriasis through activation of Stat3 or fatty acid oxidation [24, 25]. Genetic deficiency or small molecule‐mediated inhibition of FABP5 can suppress inflammation in mouse models of multiple sclerosis and ischemic stroke [26, 27]. On the contrary, in mouse models of acute lung injury and asthma, FABP5 has been shown to limit inflammation and promote anti‐inflammatory processes through peroxisome proliferator‐activated receptor β/δ (PPARβ/δ) [28, 29]. Therefore, the role of FABP5 in inflammatory processes remains controversial, as both protective and detrimental effects have been reported. In addition, as an inflammatory type of cell death, pyroptosis contributes to neuroinflammation in epilepsy, but whether FABP5 mediates pyroptosis during epilepsy progression and the underlying mechanism needs to be elucidated.

In the present study, we found that FABP5 expression was upregulated in the astrocytes in TLE patients and epilepsy models of mice and primary cells. FABP5 mediated pyroptosis and the reactive activation of astrocytes, both in vitro and in vivo. More importantly, silencing astrocytic *Fabp5* alleviated pyroptosis, inflammation, neuronal loss, and SRSs at the chronic stage of epilepsy in kainic acid (KA)‐treated epileptic mice. Mechanistically, *Fabp5* deficiency decreased lipid overload, mitochondrial damage, and the downstream cGAS‐STING signaling to play a protective role in neuroinflammation. Thus, our results reveal that astrocytic FABP5 is a critical mediator of pyroptosis in epilepsy and therefore, is a potential therapeutic target for epilepsy.

## 2 Results

### 2.1 FABP5 Is Mainly Expressed in Astrocytes and is Upregulated in TLE Patients, Epileptic Mice, and Primary Cells

**FIGURE 1:**
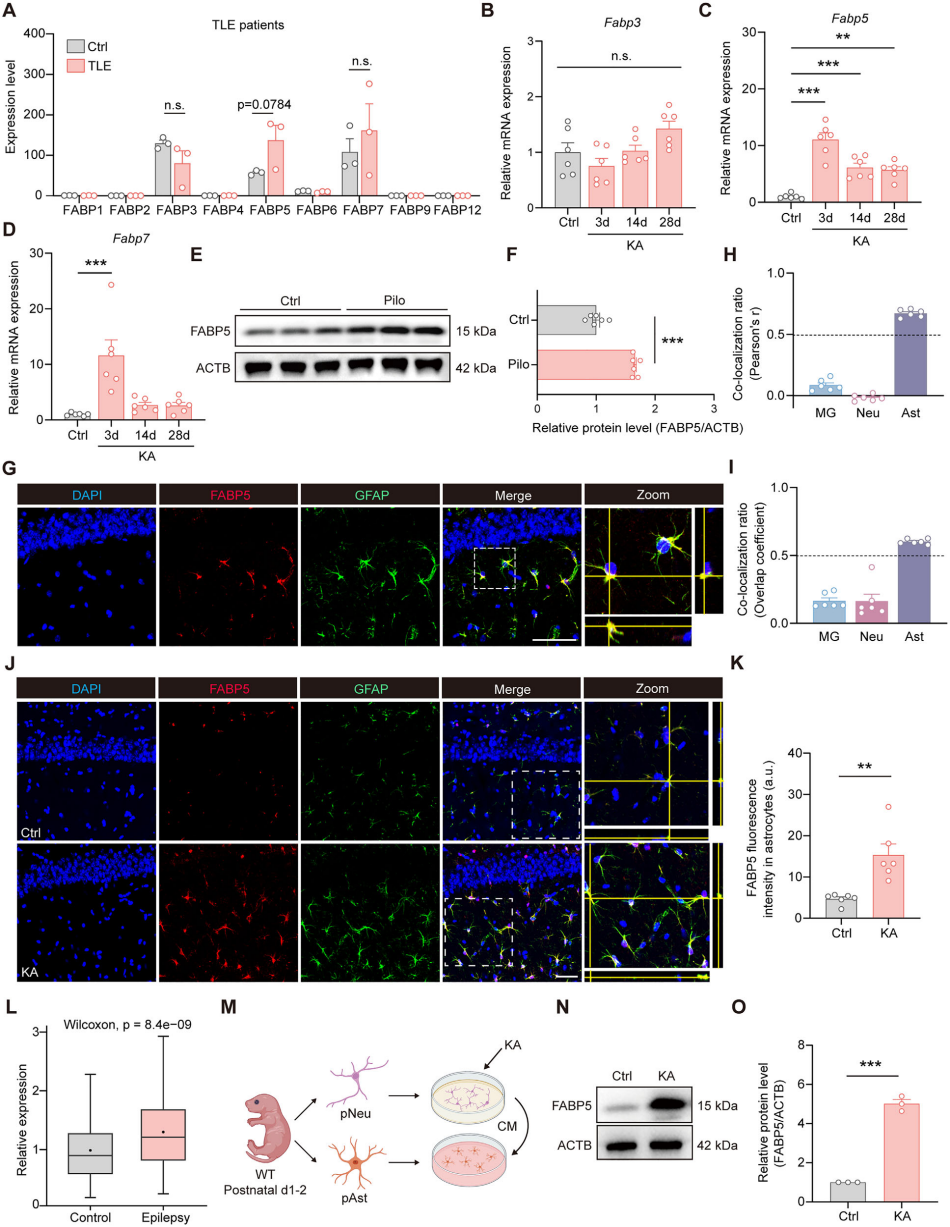
The expression of FABP5 is increased in astrocytes of TLE patients and epileptic mice. (A) The mRNA expression levels of *FABPs* in the cortex samples from TLE patients (*n* = 3 patients). (B–D) The mRNA expression levels of *Fabp3* (B), *Fabp5* (C), and *Fabp7* (D) in the hippocampus of IHKA mice at different time points (*n* = 6 mice). (E, F) Representative immunoblots (E) and statistical analysis (F) of FABP5 protein level in the hippocampus of pilocarpine‐induced epileptic mice (*n* = 6 mice). (G) Representative immunofluorescent images of GFAP (green) and FABP5 (red) double labeling in the hippocampus of IHKA mice (scale bar, 50 µm). (H, I) Co‐localization analysis of FABP5 fluorescence with GFAP, IBA1, and NeuN, respectively (*n* = 6 mice). (J, K) Representative immunofluorescent images (J) and quantification (K) of FABP5 intensity in GFAP + astrocytes in the hippocampus of IHKA mice (*n* = 6 mice; scale bar, 50 µm). (L) The mRNA expression level of *FABP5* in astrocytes from the cortex samples of TLE patients. (M) Schematic illustration of the KA‐based in vitro model used in primary astrocytes. (N, O) Representative immunoblots (N) and quantification (O) of FABP5 protein level in the primary astrocytes after CM‐KA incubation (*n* = 3 independent experiments). Data are represented as means ± SEM. Statistical analysis was performed using one‐sided Wilcoxon test (L), two‐sided unpaired Student's *t*‐tests (A, F, K, and O), and one‐way ANOVA followed by Tukey's post hoc tests (B–D). ^**^ *p* < 0.01, and ^***^ *p* < 0.001. KA, kainic acid; Pilo, pilocarpine; MG, microglia; Neu, neuron; Ast, astrocyte; CM, conditioned medium; a.u., arbitrary units; n.s., not significant.

Compared with age‐matched non‐epileptic individuals undergoing surgery for benign tumors or traumatic injury, patients with refractory TLE exhibited increased cortical mRNA levels of FABP5 and FABP7 (Figure 1A), although without statistical significance due to the sample size. In parallel, there was no significant change in *Fabp3* expression in KA‐induced epileptic mice (Figure 1B). The mRNA level of *Fabp7* was dramatically elevated only at day 3 following IHKA (Figure 1D), whereas *Fabp5* expression remained persistently upregulated from day 3 to day 28 (Figure 1C). Elevated FABP5 protein levels were also confirmed at day 14 in Pilocarpine‐induced mice (Figure 1E,F). Furthermore, immunostaining in IHKA mice was performed to delineate the specific cell types expressing FABP5. FABP5 was highly expressed in glial fibrillary acidic protein (GFAP) + astrocytes in the hippocampus following IHKA with high co‐localization ratios (Figure 1G–I), while it was barely co‐expressed in ionized calcium‐binding adapter molecule 1 (IBA1) + microglia and neuron‐specific nuclear protein (NeuN) + neurons (Figure S1A–C). These findings were in line with previous studies reporting astrocytic enrichment of FABP5 in the hippocampus [30], and further indicated that FABP5 is predominantly localized to astrocytes under epileptic conditions. In addition, epileptic mice demonstrated stronger immunoreactivity against FABP5 in astrocytes within the hippocampus compared to control mice (Figure 1J,K). Coinciding with the results in mice, reanalysis of a single‐nucleus RNA sequencing dataset of TLE patients revealed increased *FABP5* expression in astrocytes (Figure 1L and Figure S2A–F). Moreover, a previously established in vitro epilepsy model [31] was conducted to ascertain the upregulation of FABP5 (Figure 1M). Consistent with the in vivo findings, primary astrocytes displayed increased FABP5 protein expression following exposure to conditioned medium (CM) derived from KA‐treated neurons. (Figure 1N,O). Taken together, these results suggest that FABP5 is primarily expressed in astrocytes and is upregulated in patients with TLE, as well as in cellular and mouse models of epilepsy.

### 2.2 Astrocytes‐Specific Knockout of *Fabp5* Prior to IHKA Ameliorates Seizure Susceptibility and Spontaneous Recurrent Seizures in Epileptic Mice

To investigate the role of FABP5 in epilepsy, we generated an astrocyte‐specific *Fabp5* conditional knockout mouse model. The floxed allele was generated using CRISPR/Cas9‐mediated genome editing, with loxP sites inserted into non‐coding regions flanking the target exons, thereby preserving the endogenous coding sequence prior to Cre‐mediated recombination (Figure S3A). The desired insertion of the floxed allele was confirmed by PCR analysis of both the 5’ and 3’ arms (Figure S3B). FABP5 expression in the hippocampus of *Fabp5* ^fl/fl^ mice and wild‐type littermates was examined at both juvenile (4 weeks) and adult (8 weeks) stages. No significant differences in *Fabp5* mRNA expression (Figure S4A,B) or FABP5 protein levels (Figure S4C–F) were observed between the two groups at either time point. Furthermore, the spatial distribution of FABP5 in the hippocampus was comparable between *Fabp5* ^fl/fl^ and wild‐type mice, with predominant expression observed in astrocytes (Figure S4G). These results indicate that insertion of loxP sites does not affect basal FABP5 expression or its cellular distribution, thereby validating the suitability of this model for subsequent conditional knockout studies.

**FIGURE 2:**
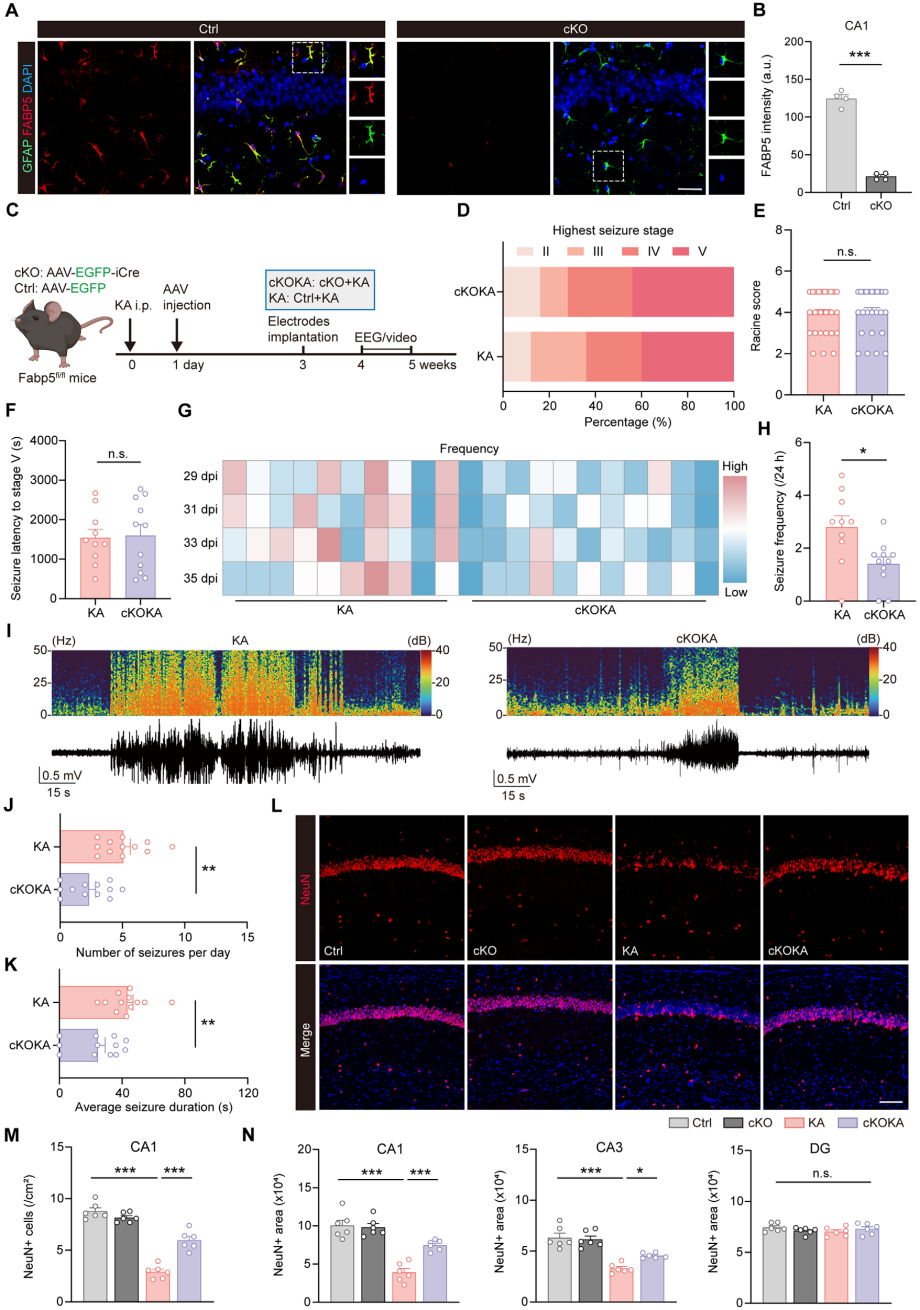
Conditional knockout of *Fabp5* in astrocytes decreases seizure activities and attenuates neuronal loss within the hippocampus in epileptic mice. (A) Representative immunofluorescent images of GFAP (green) and FABP5 (red) double labeling in the hippocampus (scale bar, 50 µm). (B) Quantification of FABP5 intensity in GFAP + astrocytes in the CA1 within the hippocampus after AAV injection (*n* = 4 mice). (C) Schematic diagram of the experimental paradigm and timeline for AAV‐mediated intervention in *Fabp5* ^fl/fl^ mice after KA induction. (D, E) Distribution of the highest seizure stage reached according to Racine scores in mice following KA induction (*n* = 25 mice). (F) Quantification of latency to the first generalized seizure (KA, *n* = 10; cKOKA, *n* = 11 mice). (G, H) Heatmap and quantification of seizure events during 4–5 weeks after KA induction (KA, *n* = 10; cKOKA, *n* = 11 mice). (I) Representative in vivo EEG recordings of 3 min at the chronic stage. (J, K) Quantitative analysis of seizure frequency and average seizure duration from in vivo EEG recordings during 4–5 weeks after KA induction (*n* = 12 days from four mice). (L) Representative images of hippocampal NeuN (red) immunofluorescence (scale bar, 100 µm). (M) Quantification of NeuN + cell number in CA1 within the hippocampus (*n* = 6 mice). (N) Quantification of NeuN+ area in the CA1, CA3, and DG regions within the hippocampus (*n* = 6 mice). Data are represented as means ± SEM. Statistical analysis was performed using two‐sided unpaired Student's *t*‐tests (B, E, F, H, J, and K) and one‐way ANOVA followed by Tukey's post hoc tests (M, N). ^*^ *p* < 0.05, ^**^ *p* < 0.01, and ^***^ *p* < 0.001. n.s., not significant; cKO, conditional knockout.

An adeno‐associated virus (AAV) encoding the gfaABC1D promoter‐driven iCre (cKO group) or a control AAV (Ctrl group) was injected into the Cornu Ammonis 1 (CA1) and dentate gyrus (DG) regions of *Fabp5* ^fl/fl^ mice to specifically reduce FABP5 expression in astrocytes. (Figure S5A,B). AAV was expressed strictly in astrocytes but not microglia and neurons (Figure S5C–H). Immunostaining and western blot confirmed a significant reduction in FABP5 protein expression in the hippocampus of cKO mice (Figure 2A,B and Figure S6A–F). Together, these findings demonstrate that AAV‐Cre‐mediated recombination efficiently and specifically deletes *Fabp5* in astrocytes, providing a reliable model for investigating the functional role of astrocytic FABP5 in epilepsy.

Mice underwent IHKA 3 weeks after AAV injection (Figure S7A), and behavioral seizures were evaluated during the acute phase using the Racine scale. Mice with astrocytic *Fabp5* deletion (cKOKA group) displayed a lower ratio reaching stage IV and stage V relative to control AAV‐injected mice (KA group) (Figure S7B), although the overall Racine score did not differ significantly between groups (Figure S7C). Importantly, seizure latency to stage V where mice experienced a generalized seizure, was markedly prolonged in cKOKA mice, indicating decreased seizure susceptibility (Figure S7D). Additionally, cKOKA mice exhibited reduced spontaneous recurrent seizures (SRSs) frequency at the chronic phase of epilepsy (Figure S7E,F). These findings indicate that astrocytic FABP5 contributes to seizure susceptibility and SRSs in mice.

**TABLE 1:** Primer sequences.

Gene	Primer	Sequence
*Fabp3*	Forward	AGAGTTCGACGAGGTGACAGCA
	Reverse	TTGTCTCCTGCCCGTTCCACTT
*Fabp5*	Forward	GACGACTGTGTTCTCTTGTAACC
	Reverse	TGTTATCGTGCTCTCCTTCCCG
*Fabp7*	Forward	CAGTCAGGAAGGTGGCAAAGTG
	Reverse	GCTTGTCTCCATCCAACCGAAC
*Gfap*	Forward	AGATTCGCACTCAATACGAGG
	Reverse	CTGTGAGGTCTGCAAACTTAG
*Il‐1β*	Forward	GCAGAGCACAAGCCTGTCTTCC
	Reverse	ACCTGTCTTGGCCGAGGACTAAG
*C3*	Forward Reverse	CGCAACGAACAGGTGGAGATCA CTGGAAGTAGCGATTCTTGGCG
*Isg15*	Forward	CATCCTGGTGAGGAACGAAAGG
	Reverse	CTCAGCCAGAACTGGTCTTCGT
*Irf7*	Forward	CCTCTGCTTTCTAGTGATGCCG
	Reverse	CGTAAACACGGTCTTGCTCCTG
*Cxcl10*	Forward	ATCATCCCTGCGAGCCTATCCT
	Reverse	GACCTTTTTTGGCTAAACGCTTTC
*β‐actin*	Forward	CTACCTCATGAAGATCCTGACC
	Reverse	CACAGCTTCTCTTTGATGTCAC
*mt‐Nd1*	Forward	CAAACACTTATTACAACCCAAGAACA
	Reverse	TCATATTATGGCTATGGGTCAGG
*mt‐Nd4*	Forward	AACGGATCCACAGCCGTA
	Reverse	AGTCCTCGGGCCATGATT
*nuc‐Tert*	Forward	CTAGCTCATGTGTCAAGACCCTCTT
	Reverse	GCCAGCACGTTTCTCTCGTT

### 2.3 Conditional Knockout of *Fabp5* in Astrocytes Ameliorates Seizure Activities and Neuronal Loss in Epileptic Mice

Our preceding data indicated that *Fabp5* deficiency attenuated SRSs in epileptic mice. However, *Fabp5* deletion prior to IHKA may influence the initial insult used to trigger epileptogenesis. The frequency of SRSs correlates directly with the severity of the status epilepticus, which makes it unclear whether *Fabp5* deletion attenuated SRSs by affecting epilepsy development (epileptogenesis) or seizure susceptibility [32]. Therefore, a parallel experiment was conducted (Figure 2C), in which AAV was injected one day after KA induction to minimize the impact of different seizure susceptibility on chronic‐phase SRSs severity. Lesions induced by the systemic administration of KA are comparable to those caused by intracerebral administration [33]. To minimize brain damage from two consecutive days of surgery, KA was administered intraperitoneally in this experiment. KA‐injected mice were randomly assigned to two groups, and no differences in Racine score distribution or latency to the first generalized seizures were identified between cKOKA and KA groups (Figure 2D–F).

Given that SRSs generally reach a maximum at 4–6 weeks after KA induction and subsequently decline in C57BL6/J mice [34, 35], video‐EEG recordings were employed 4 weeks post‐induction. The frequency of spontaneous behavioral convulsive seizures was notably dampened when *Fabp5* was knocked out in astrocytes (Figure 2G,H). KA‐treated mice developed spontaneous electrographic seizures lasting for at least 1 week, as evidenced by the high‐frequency local field potential oscillations in EEG traces, which were eminently inhibited in cKOKA mice (Figure 2I). Quantitative analysis revealed that cKOKA mice exhibited reduced seizure frequency and shorter average seizure duration (Figure 2J,K), indicating alleviated SRSs. Furthermore, astrocytic *Fabp5* deletion markedly mitigated neuronal loss in the CA1 and CA3 regions of epileptic mice, as defined by NeuN immunostaining in the hippocampus (Figure 2L–N and Figure S8A). Taken together, these results suggest that astrocytic FABP5 contributes to the progression of epilepsy. Specifically, astrocyte‐specific *Fabp5* knockout is sufficient to attenuate SRSs and neuron damage in KA‐induced epileptic mice.

### 2.4 FABP5 Regulates Pyroptosis and the Reactive Activation of Astrocytes In Vitro

**FIGURE 3:**
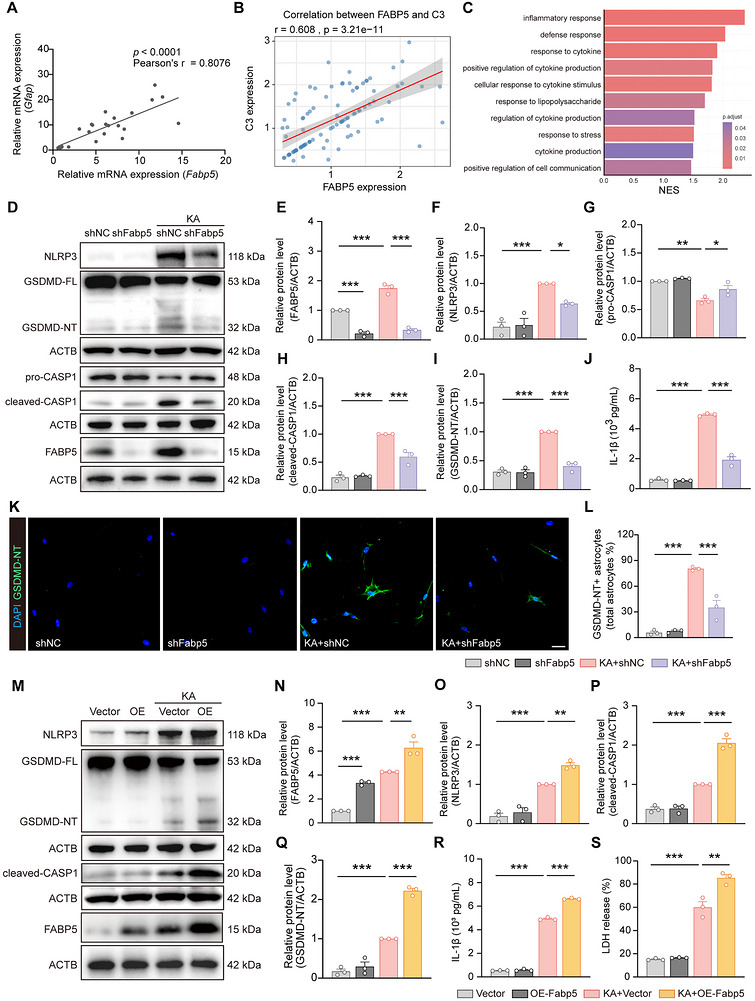
FABP5 regulates pyroptosis and the reactive activation of astrocytes in vitro. (A) Correlation analysis between *Gfap* and *Fabp5* mRNA expression levels in the hippocampus of IHKA mice (*n* = 24 mice). (B) Correlation analysis between *C3* and *FABP5* mRNA expression levels in astrocytes from TLE patients. (C) The upregulated pathways in *FABP5*‐expressing astrocytes relative to non‐expressing astrocytes from TLE patients. (D–I) Representative immunoblots (D) with statistical analysis (E–I) of FABP5 (E), NLRP3 (F), pro‐CASP1 (G), cleaved‐CASP1 (H), and GSDMD‐NT (I) protein levels in CM‐KA‐primed primary astrocytes transduced with *Fabp5*‐shRNA lentivirus or negative lentivirus (*n* = 3 independent experiments). (J) The protein level of IL‐1β detected by ELISA in the cell supernatants from primary astrocytes (*n* = 3 independent experiments). (K, L) Representative immunofluorescent images (K) and quantification (L) of GSDMD‐NT (green) labeled primary astrocytes (*n* = 3 independent experiments; scale bar, 100 µm). (M‐Q) Representative immunoblots (M) with statistical analysis (N–Q) of FABP5 (N), NLRP3 (O), cleaved‐CASP1 (P), and GSDMD‐NT (Q) protein levels in CM‐KA‐primed primary astrocytes transduced with *Fabp5*‐overexpressing lentivirus or vector (*n* = 3 independent experiments). (R) The protein level of IL‐1β detected by ELISA in the cell supernatants from primary astrocytes (*n* = 3 independent experiments). (S) Level of LDH release in the cell supernatants from primary astrocytes (*n* = 3 independent experiments). Data are represented as means ± SEM. Statistical analysis was performed using one‐way ANOVA followed by Tukey's post hoc tests. ^*^ *p* < 0.05, ^**^ *p* < 0.01, and ^***^ *p* < 0.001. KA, kainic acid; NC, negative control; OE, overexpression; NES, normalized enrichment score.

Several studies have indicated the participation of FABP5 in the inflammatory processes. In a recent study, *Fabp5* was designated as one of the signatures of reactive astrocytes within the stroke infarct site, where inflammation responses may be dramatic [36]. In the present study, *Fabp5* mRNA levels correlated positively with the reactive astrocyte marker *Gfap* in the hippocampus from IHKA mice (Figure 3A). Similarly, in the astrocytes from TLE patients, *FABP5* mRNA levels were positively correlated with *GFAP* and with the inflammatory astrocytic markers, including complement 3 (*C3*) and *NFKB1* (Figure 3B and Figure S9A,B). Moreover, we analyzed the differences in enriched biological pathways between *FABP5*‐expressing and non‐expressing astrocytes in TLE patients. Gene set enrichment analysis (GSEA) employing Gene Ontology (GO) gene sets showed that pathways related to inflammatory responses were significantly upregulated in *FABP5*‐expressing astrocytes (Figure 3C and Figure S9C–F), suggesting a strong association between astrocytic *FABP5* expression and inflammation under epileptic conditions.

To assess the effects of FABP5 on astrocyte activation and pyroptosis – a form of inflammatory cell death, primary astrocytes were transduced with lentivirus carrying *Fabp5*‐specific shRNA (sh*Fabp5*) or a negative control (shNC), followed by CM‐KA incubation. Knockdown efficiency was validated by qRT‐PCR and western blot analysis (Figure S10A–C). Under epileptic stress, astrocytes exhibited marked morphological alterations compared to control groups, characterized by retraction of cellular processes, occasional bleb‐like structures, and a more contracted, thickened cell body. Notably, these changes were partially alleviated by *Fabp5* knockdown (Figure S10D). Pyroptosis is characterized by cell swelling, disorganized intracellular architecture, and loss of membrane integrity. Accordingly, transmission electron microscopy (TEM) was performed to evaluate ultrastructural changes. Astrocytes in the control groups exhibited intact morphology with preserved organelles, whereas CM‐KA‐treated cells displayed pronounced cellular damage, including cytoplasmic swelling, organelle disruption, and partial loss of membrane integrity. Importantly, these alterations were partially alleviated by *Fabp5* knockdown. (Figure S10E,F). However, accumulating evidence indicated that, unlike macrophages, which were primarily used to study pyroptosis, other cell types, including neutrophils [37, 38] and intestinal epithelial cells [39], lacked typical features and instead exhibited distinct morphological characteristics. Therefore, morphological observations alone cannot reliably define pyroptotic cell death in astrocytes [40]. Accordingly, to further determine whether pyroptosis is involved, we next examined key pyroptosis‐related markers, including GSDMD cleavage, by Western blot analysis.

Increased NLRP3, cleaved‐CASP1, and GSDMD‐NT levels, along with decreased pro‐CASP1 expression, confirmed that pyroptosis occurred under epileptic conditions. Conversely, *Fabp5* knockdown markedly reduced the increased levels of pyroptosis‐related proteins (Figure 3D–I). Besides, data from ELISA, qRT‐PCR, and lactate dehydrogenase (LDH) assays demonstrated that the release of IL‐1β and LDH, as well as the expression of astrocytic activation markers *C3* and *Gfap*, were highly induced after CM‐KA incubation, whereas *Fabp5* knockdown partly reversed these effects (Figure 3J and Figure S10G–I). In concert with the above results, parallel alteration of GSDMD‐NT in diverse groups was further confirmed by immunostaining (Figure 3K,L). To further probe into the potential role of FABP5 in pyroptosis, primary astrocytes were transduced with a *Fabp5*‐overexpressing (OE‐*Fabp5*) lentivirus (Figure S11A). In contrast to the knockdown experiment, overexpression further exacerbated pyroptosis, as indicated by elevated levels of NLRP3, cleaved‐CASP1, GSDMD‐NT, secreted IL‐1β, and LDH (Figure 3M–S). Similarly, *Gfap* and *C3* expression levels were strikingly increased in *Fabp5*‐overexpressing astrocytes (Figure S11B,C). Collectively, these outcomes reveal that FABP5 promotes pyroptosis and the inflammatory activation of astrocytes.

### 2.5 Astrocytic *Fabp5* Deficiency Alleviated Neuroinflammation in Epileptic Mice by Downregulating Pyroptosis

**FIGURE 4:**
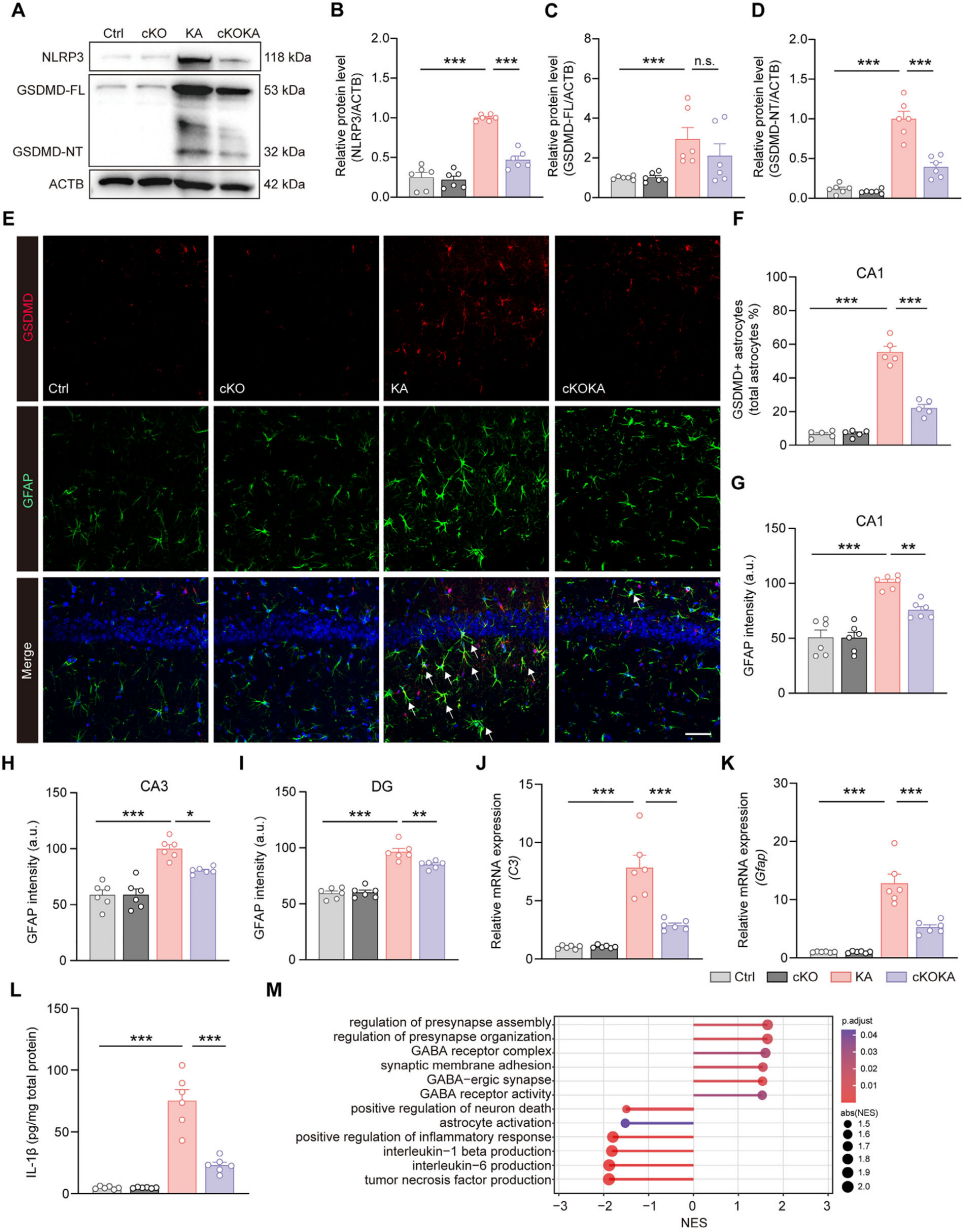
Astrocytic *Fabp5* deficiency alleviates neuroinflammation in epileptic mice via downregulation of pyroptosis. (A‐D) Representative immunoblots (A) with statistical analysis (B–D) of NLRP3 (B), GSDMD‐FL (C), and GSDMD‐NT (D) protein levels in *Fabp5* ^fl/fl^ mice injected with AAV after KA induction (*n* = 6 mice). (E) Representative immunofluorescent images of GFAP (green) and GSDMD (red) double labeling in the hippocampus (scale bar, 50 µm). (F–I) Ratio of GSDMD + astrocytes in total astrocytes (F) and quantification of GFAP intensity in the CA1 (G), CA3 (H), and DG (I) regions within the hippocampus (*n* = 6 mice). (J, K) The mRNA expression levels of *C3* (J) and *Gfap* (K) in the hippocampus (*n* = 6 mice). (L) The protein level of IL‐1β detected by ELISA in the hippocampus (*n* = 6 mice). (M) The up‐ and down‐regulated pathways related to epilepsy and neuroinflammation in the hippocampus from cKOKA mice relative to KA mice. Data are represented as means ± SEM. Statistical analysis was performed using one‐way ANOVA followed by Tukey's post hoc tests. ^*^ *p* < 0.05, ^**^ *p* < 0.01, and ^***^ *p* < 0.001. n.s., not significant; cKO, conditional knockout; NES, normalized enrichment score.

To further verify in vitro findings that *Fabp5* knockdown attenuates pyroptosis, we examined pyroptotic activity in the hippocampus of diverse groups. Similar outcomes were obtained in vivo. Elevated pyroptosis was detected within the hippocampus of KA‐induced mice, and this increase was restored by astrocytic *Fabp5* deletion (Figure 4A–D). Astrocytes are activated within 1–2 days after the induction of status epilepticus, and reactive astrocytes may be detectable for over 3 to 4 months [41]. Robust activation of astrocytes, especially GSDMD‐positive astrocytes, was observed in the CA1, CA3, and DG regions of KA mice. This activation was reversed by *Fabp5* conditional knockout, as measured by immunofluorescence (Figure 4E–I). Meanwhile, qRT‐PCR analysis showed that the increased mRNA expression of *Gfap* and *C3* was also abrogated in cKOKA mice (Figure 4J,K), implying alleviated astrocytic activation. Reactive astrocytes are neurotoxic and serve to further release pro‐inflammatory cytokines (e.g., IL‐1β, IL‐6, and TNF‐α), which recruit additional inflammatory cells and result in neuronal hyperexcitability and damage [42]. Accordingly, ELISA revealed a marked reduction in IL‐1β protein levels in cKOKA mice (Figure 4L).

RNA sequencing was performed with total RNAs extracted from the hippocampi of cKOKA mice and KA mice. Significant transcriptomic shifts were evident in the hippocampus between cKOKA and KA group (Figure S12A,B). Downregulated pathways in cKOKA mice were enriched in biological processes relevant to glial activation, neuronal death, and cytokines and chemokines signaling, including NLRP and NF‐*k*B pathways (Figure 4M and Figure S12C–G), consistent with aforementioned data concerning hippocampal pyroptosis and inflammation. Conversely, upregulated pathways were related to synaptic structure and function, as well as GABA‐ergic synapse and receptor (Figure 4M). Together, these data indicate that astrocytic FABP5 exerts pathological effects in the context of TLE by promoting pyroptosis and neuroinflammation.

### 2.6 Conditional Knockout of *Fabp5* in Astrocytes Attenuates cGAS‐STING Signaling Activation in Epileptic Mice

**FIGURE 5:**
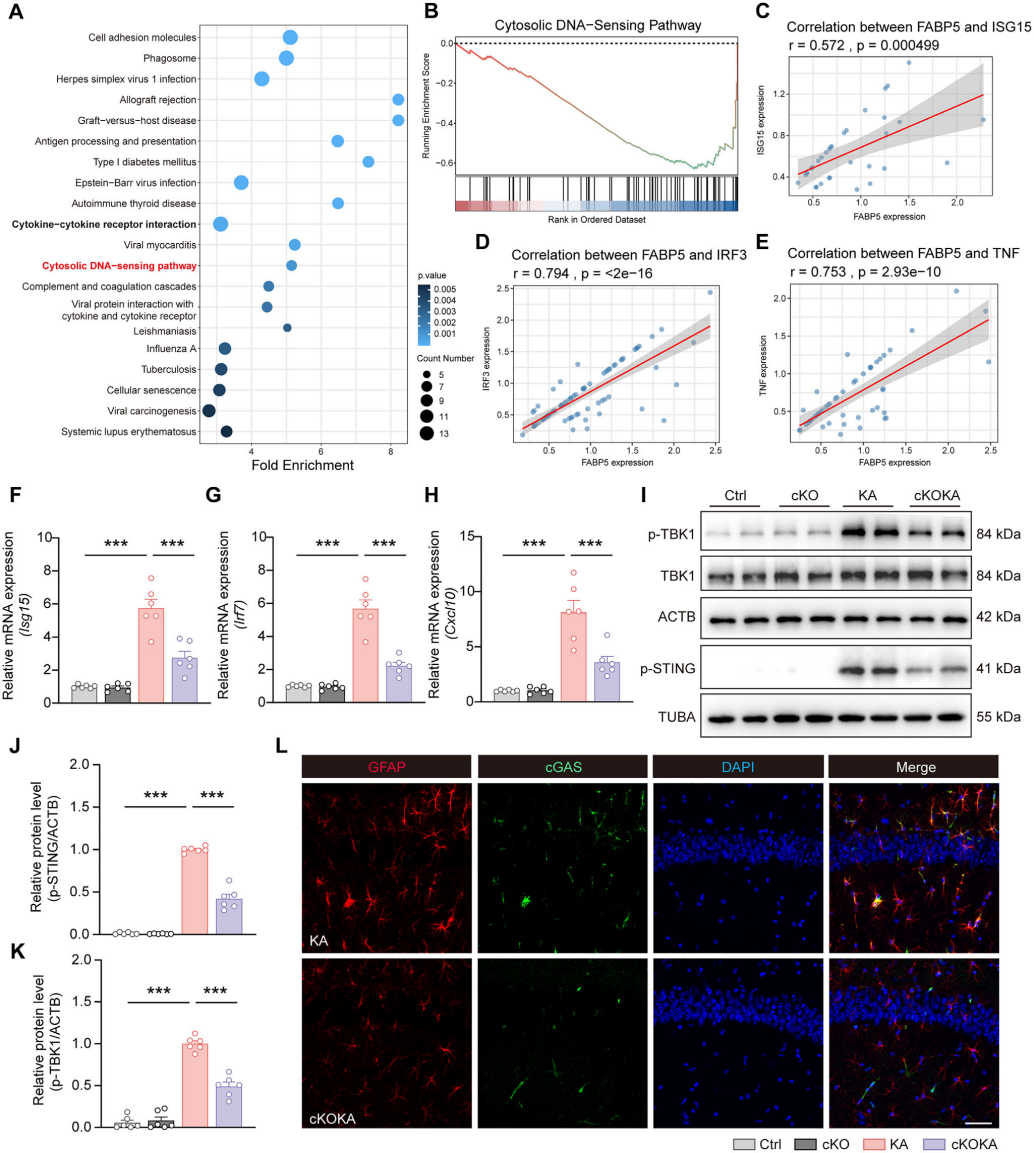
Conditional knockout of *Fabp5* in astrocytes attenuates cGAS‐STING signaling activation in epileptic mice. (A) KEGG analysis of identified DEGs showing the top 20 enriched pathways between cKOKA mice and KA mice. (B) GSEA showing the downregulation of the cytosolic DNA‐sensing pathway in cKOKA mice relative to KA mice. (C–E) Correlation of the mRNA expression levels between *FABP5* and *ISG15* (C), *IRF3* (D), and *TNF* (E) in the astrocytes from TLE patients. (F–H) The mRNA expression levels of *Isg15* (F), *Irf7* (G), and *Cxcl10* (H) in the hippocampus (*n* = 6 mice). (I–K) Representative immunoblots (I) with statistical analysis (J‐K) of p‐STING (Ser365) and p‐TBK1(Ser172) protein levels in *Fabp5* ^fl/fl^ mice injected with AAV after KA induction (*n* = 6 mice). (L) Representative immunofluorescent images of GFAP (red) and cGAS (green) double labeling in the hippocampus (scale bar, 50 µm). Data are represented as means ± SEM. Statistical analysis was performed using one‐way ANOVA followed by Tukey's post hoc tests. ^***^ *p* < 0.001. cKO, conditional knockout.

It has become evident that FABP5 is a critical mediator of inflammatory processes through metabolic or signaling mechanisms [20]. To uncover the potential downstream targets of FABP5 in mediating astrocyte pyroptosis, Kyoto Encyclopedia of Genes and Genomes (KEGG) annotation was conducted using differentially expressed genes (DEGs) between cKOKA and KA groups. Strikingly, the cytosolic DNA‐sensing pathway ranked among the top 20 enriched pathways (Figure 5A). GSEA demonstrated that the cytosolic DNA‐sensing pathway, always referring to cGAS‐STING axis, was markedly downregulated in cKOKA mice (Figure 5B). cGAS‐STING pathway is a critical driver of immune responses involving robust interferon (IFN) activation against microbial infection [43] and has recently been discovered to trigger aberrant sterile inflammation, neurotoxicity, and cognitive impairment in neurodegenerative diseases [44]. In TLE patients, *FABP5*‐expressing astrocytes exhibited high mRNA levels of mediators involved in IFN response or its additional pathways. Importantly, *FABP5* level was positively correlated with interferon‐stimulated gene 15 (*ISG15*), interferon regulatory factor 3 (*IRF3*), and tumor necrosis factor (*TNF*), in astrocytes of TLE patients (Figure 5C–E).

In the present experiment, RNA‐seq results were confirmed by qRT‐PCR of selected genes involved in cGAS‐STING pathway in the hippocampus (Figure 5F–H). Consistent with the mRNA assay, protein levels of phospho‐STING (Ser365) and phospho‐TBK1 (Ser172) were markedly augmented in the hippocampus from KA mice, and this upregulation was abolished by *Fabp5* deletion (Figure 5I–K). Additionally, double immunostaining further verified an evident reduction of cGAS expression in astrocytes within the CA1 region in cKOKA mice (Figure 5L). Collectively, the above findings imply that cGAS‐STING signaling cascade functions as a downstream effector of FABP5 during epilepsy progression.

### 2.7 FABP5 Contributes to Astrocytic Pyroptosis via Mitochondria Damage and cGAS‐STING Signaling Activation

**FIGURE 6:**
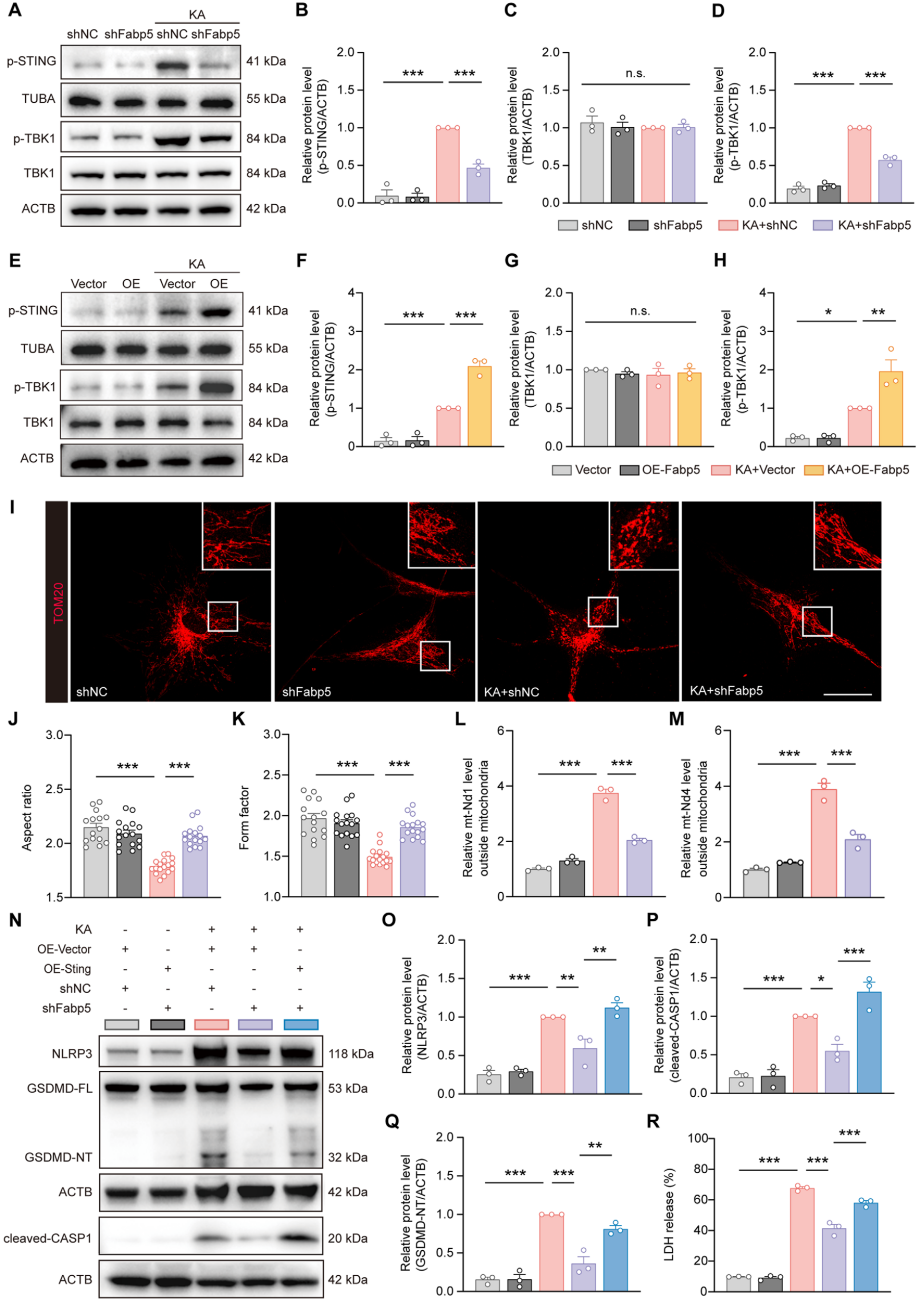
FABP5 mediates astrocytes' pyroptosis via mitochondria injury and cGAS‐STING signaling pathway. (A–D) Representative immunoblots (A) with statistical analysis (B–D) of p‐STING (B), TBK1 (C), and p‐TBK1 (D) protein levels in CM‐KA‐primed primary astrocytes transduced with *Fabp5*‐shRNA lentivirus or negative lentivirus (*n* = 3 independent experiments). (E–H) Representative immunoblots (E) with quantification (F–H) of p‐STING (F), TBK1 (G), and p‐TBK1 (H) protein levels in CM‐KA‐primed primary astrocytes transduced with *Fabp5*‐overexpressing lentivirus or vector (*n* = 3 independent experiments). (I–K) Representative immunofluorescent images (I) of TOM20 (red) labeled primary astrocytes and quantification (J, K) of aspect ratio and form factor (*n* = 15–16 views containing 1–2 cells each from three independent experiments; scale bar, 50 µm). (L, M) Cytosolic mtDNA detected by qRT‐PCR using *Nd1* and *Nd4* primers in CM‐KA‐primed primary astrocytes transduced with *Fabp5*‐shRNA lentivirus or negative lentivirus. Nuclear DNA in whole‐cell lysate detected by the *Tert* was used for standardization. (*n* = 3 independent experiments). (N–Q) Representative immunoblots (N) with quantification (O–Q) of NLRP3 (O), cleaved‐CASP1 (P), and GSDMD‐NT (Q) protein levels in CM‐KA‐primed *Fabp5*‐knockdown primary astrocytes transduced with *Sting*‐overexpressing lentivirus or vector (*n* = 3 independent experiments). (R) Levels of LDH release in the cell supernatants from primary astrocytes (*n* = 3 independent experiments). Data are represented as means ± SEM. Statistical analysis was performed using one‐way ANOVA followed by Tukey's post hoc tests. ^*^ *p* < 0.05, ^**^ *p* < 0.01, and ^***^ *p* < 0.001. KA, kainic acid; NC, negative control; OE, overexpression; n.s., not significant.

Following these findings in vivo, we next validated whether FABP5 affects cGAS‐STING signaling activation in primary astrocytes. The levels of p‐TBK1 and p‐STING were obviously increased after CM‐KA incubation, with no change in total TBK1. *Fabp5* knockdown strongly abolished the upregulation of these proteins (Figure 6A–D), whereas *Fabp5* overexpression further enhanced cGAS‐STING axis activation (Figure 6E–H). Under sterile conditions, activation of the cGAS‐STING pathway is primarily driven by endogenous DNA. Notably, mitochondrial DNA (mtDNA) released into the cytosol following mitochondrial perturbation represents a major and well‐established trigger of cGAS activation [45]. CM‐KA‐primed astrocytes demonstrated a more punctate mitochondrial morphology as evidenced by TOM20 immunofluorescence, implying eminent mitochondria damage. Contrarily, *Fabp5* knockdown noticeably ameliorated mitochondria damage, showing increased aspect ratio and form factor in comparison with CM‐KA group (Figure 6I–K). Moreover, cytosolic release of mtDNA was strikingly enhanced in CM‐KA‐induced astrocytes, and this enhancement was restored by *Fabp5* knockdown (Figure 6L,M), consistent with its inhibitory effect on cGAS‐STING signaling.

To investigate whether FABP5 mediates pyroptosis through cGAS‐STING pathway, *Sting*‐overexpressing (OE‐*Sting*) lentivirus was introduced into *Fabp5*‐deficient astrocytes to enforce STING expression. Immunoblot analysis confirmed increased protein expression of STING (Figure S13A,B). Under basal conditions, *Sting* overexpression did not directly trigger pyroptosis in astrocytes, as no differences were observed in the protein levels of NLRP3, GSDMD‐FL, and GSDMD‐NT (Figure S13A–D). Following CM‐KA treatment, cGAS‐STING signaling was strongly activated in *Sting*‐overexpressing astrocytes (Figure S13E–H). While knockdown of *Fabp5* led to a remarkable downregulation of pyroptosis‐related proteins (NLRP3, cleaved‐CASP1, and GSDMD‐NT) and decreased LDH release, *Sting* overexpression noticeably rescued these effects (Figure 6N–R). In addition, *Sting* overexpression partially counteracted the reduction in astrocytic activation by *Fabp5* knockdown (Figure S13I,J). Together, these results indicate that FABP5 promotes mitochondria damage, mtDNA release, and the subsequent activation of cGAS‐STING signaling, thereby unleashing pyroptosis and activation of astrocytes in epilepsy.

### 2.8 Inhibition of STING Attenuates Pyroptosis, Astrocyte Activation, and Seizure Activities in IHKA Mice

Our previous results demonstrated that FABP5 contributes to neuroinflammation and epilepsy through the cGAS‐STING signaling pathway. To determine whether STING inhibition can reproduce the anti‐epileptic effects of FABP5 inhibition, we used an inhibitor specifically targeting mouse STING (C‐176). Under physiological conditions, STING inhibition did not affect basal pyroptosis or inflammatory status in the hippocampus. Western blot analysis showed that administration of C‐176 had no significant effect on the protein levels of NLRP3 and GSDMD‐FL in the hippocampus (Figure S14A–C). Consistently, immunofluorescence staining and qPCR analysis revealed no evident astrocyte activation in the hippocampus of C‐176‐treated mice and vehicle controls (Figure S14D–G). Moreover, overall hippocampal inflammatory levels remained comparable between the two groups (Figure S14H). These results suggest that STING signaling is largely inactive under basal conditions and that its inhibition does not disrupt physiological homeostasis.

**FIGURE 7:**
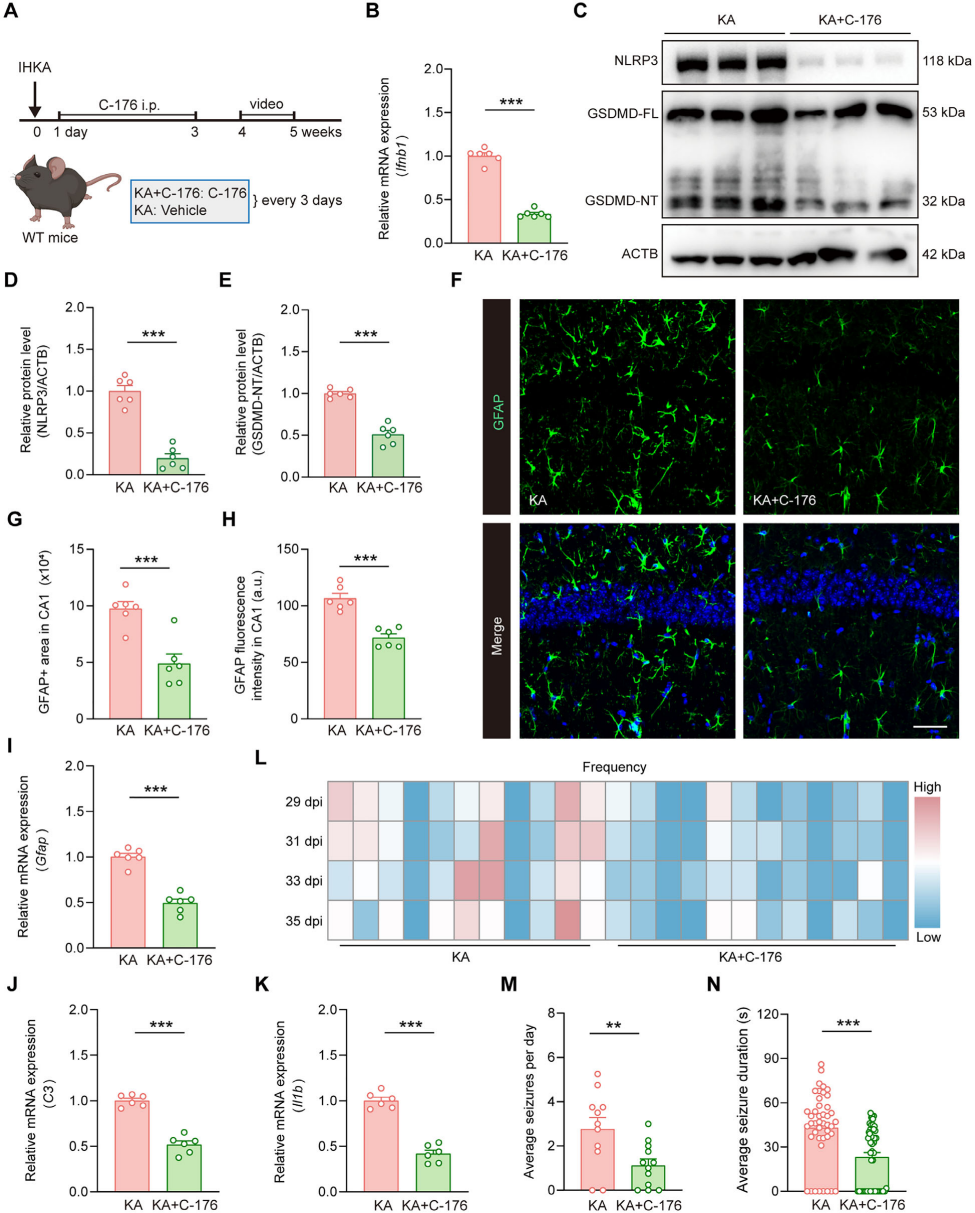
Inhibition of STING by C‐176 attenuates pyroptosis, astrocyte activation, and seizure activities during the chronic stage of epilepsy. (A) Diagram of the experimental paradigm and timeline for C‐176 or vehicle intervention in WT mice following IHKA. (B) Analysis of *Ifnb1* mRNA expression in the hippocampus (*n* = 6 mice). (C–E) Representative immunoblots (C) with statistical analysis of NLRP3 (D) and GSDMD‐NT (E) protein levels in mice injected with C‐176 or vehicle after IHKA (*n* = 6 mice). (F) Representative immunofluorescent images of GFAP (green) labeling in the hippocampus (scale bar, 50 µm). (G, H) Quantification of GFAP + area and intensity in the CA1 within the hippocampus (*n* = 6 mice). (I–K) Analysis of mRNA levels of *Gfap* (I), *C3* (J), and *Il1b* (K) in the hippocampus (*n* = 6 mice). (L, M) Heatmap and quantification of seizure frequency during 4–5 weeks after IHKA (KA, *n* = 11; KA + C‐176, *n* = 12 mice). (N) Quantification of average seizure duration during 4–5 weeks after IHKA (KA, *n* = 44 days from 11 mice; KA+C‐176, *n* = 48 days from 12 mice). Data are represented as means ± SEM. Statistical analysis was performed using two‐sided unpaired Student's *t* ‐tests (B, D, E, G—K, and M) and the Mann‐Whitney test (N). ^**^ *p* < 0.01, and ^***^ *p* < 0.001. WT, wildtype.

We next investigated whether STING inhibition modulates pyroptosis and neuroinflammation in the KA‐induced epilepsy model. Mice were injected with C‐176 intraperitoneally every 3 days until 3 weeks after IHKA (Figure 7A). Inhibition of STING signaling was validated by qRT‐PCR (Figure 7B). Mice treated with C‐176 demonstrated reduced levels of pyroptosis‐related proteins, including NLRP3 and GSDMD‐NT (Figure 7C–E), consistent with the findings in *Fabp5* conditional knockout mice. Moreover, C‐176 markedly attenuated astrocyte activation in the hippocampus, as shown by immunofluorescence staining against GFAP (Figure 7F–H) and qRT‐PCR analysis of *Gfap* and *C3* (Figure 7I,J). The mRNA level of *Il1b* was significantly reduced in C‐176‐treated mice (Figure 7K), indicating attenuated neuroinflammation. Similar to astrocyte‐specific *Fabp5* knockout, C‐176 notably reduced spontaneous behavioral convulsive seizures, as evidenced by decreased frequency and average duration of seizures (Figure 7L–N). Collectively, these results indicate that STING inhibition attenuates pyroptosis and SRSs during the chronic stage of epilepsy.

### 2.9 Inhibition of *Fabp5* Attenuated Lipid Accumulation and Mitochondrial Dysfunction in Astrocytes in Epileptic Models

Our data demonstrate that FABP5 promotes cGAS‐STING activation and downstream pyroptosis; however, the upstream mechanisms linking FABP5 to cytosolic DNA sensing remain to be elucidated. Notably, *Fabp5* knockdown significantly reduced mitochondrial damage and the release of mtDNA, a major endogenous activator of cGAS, suggesting that mitochondrial dysfunction may underlie FABP5‐mediated pathway activation. Given that FABP5 is a lipid chaperone involved in intracellular fatty acid trafficking and lipid partitioning [22], we hypothesized that aberrant lipid accumulation may impose excessive lipid burden on mitochondria under pathological conditions, thereby inducing mitochondrial stress and damage, and ultimately triggering cGAS‐STING activation. Therefore, we next investigated lipid accumulation and mitochondrial alterations under epileptic conditions.

**FIGURE 8:**
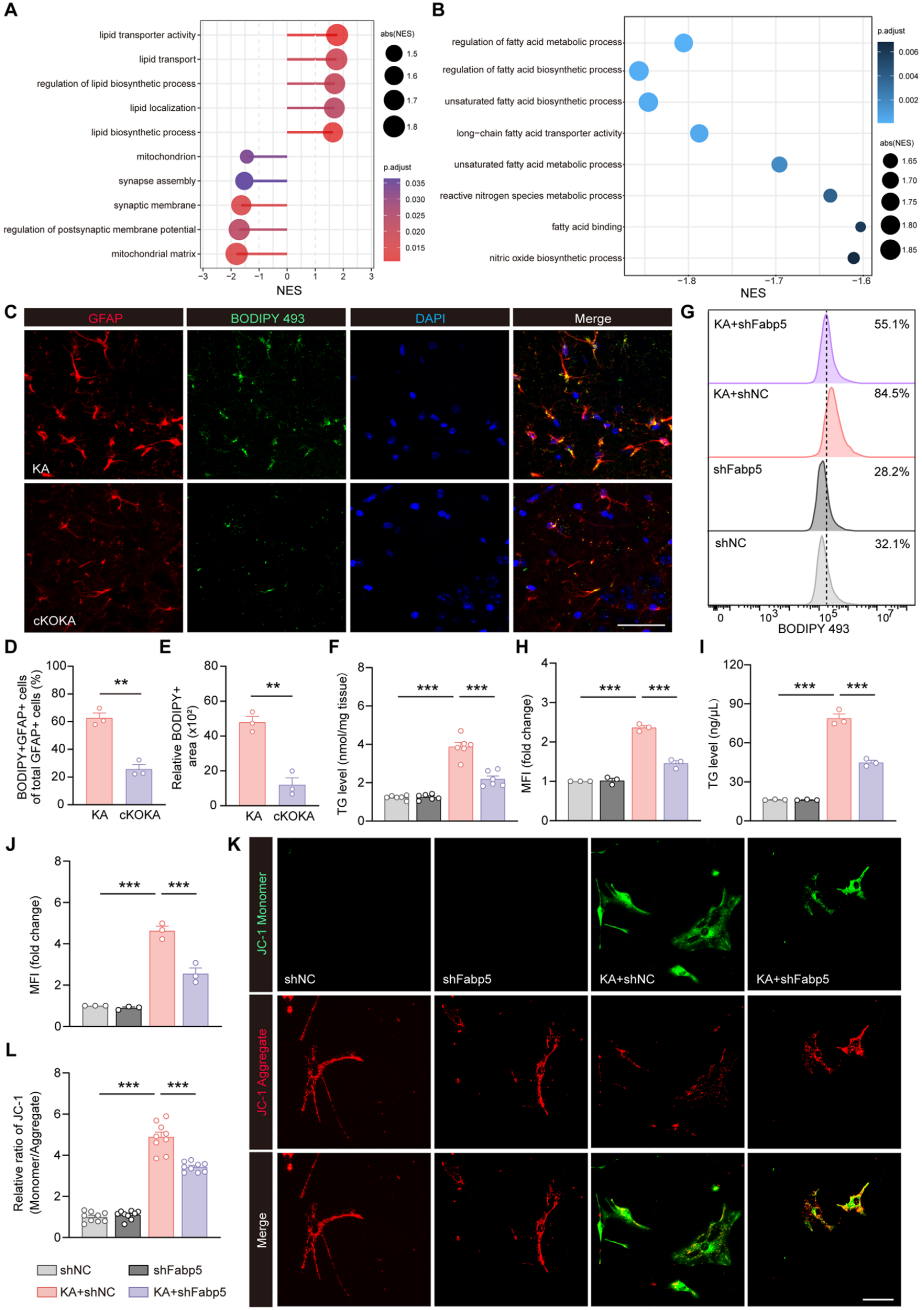
*Fabp5* deficiency attenuates lipid overload and mitochondrial dysfunction in astrocytes. (A) The upregulated pathways related to lipid biological processes in *FABP5*‐expressing astrocytes relative to non‐expressing astrocytes from TLE patients. (B) The down‐regulated pathways related to fatty acid and ROS biological processes in the hippocampus from cKOKA mice relative to KA mice. (C) Representative immunofluorescent images of GFAP (red) and BODIPY (green) double labeling in the hippocampus from cKOKA or KA mice (scale bar, 50 µm). (D, E) Ratio of BODIPY+ astrocytes in total astrocytes (D) and quantification of BODIPY + area (E) in the hippocampus (*n* = 3 mice). (F) Quantification of hippocampal triglyceride levels across groups (*n* = 6 mice). (G, H) Flow cytometric analysis (G) and quantification (H) of BODIPY levels in CM‐KA‐primed primary astrocytes transduced with *Fabp5*‐shRNA lentivirus or negative lentivirus (*n* = 3 independent experiments). (I) Triglyceride quantification in CM‐KA‐primed primary astrocytes transduced with *Fabp5*‐shRNA lentivirus or negative lentivirus (*n* = 3 independent experiments). (J) Flow cytometric analysis of MitoSOX fluorescence in CM‐KA‐primed primary astrocytes transduced with *Fabp5*‐shRNA lentivirus or negative lentivirus (*n* = 3 independent experiments). (K, L) Representative JC‐1 staining images (K) and quantitative analysis (L) of JC‐1 green/red fluorescence ratio (*n* = 9 views from three independent experiments; scale bar, 50 µm). Data are represented as means ± SEM. Statistical analysis was performed using two‐sided unpaired Student's *t*‐tests (D, E) and one‐way ANOVA followed by Tukey's post hoc tests (F, H‐J, L). ^**^ *p* < 0.01, and ^***^ *p* < 0.001. KA, kainic acid; NC, negative control; cKO, conditional knockout; MFI, mean fluorescence intensity; NES, normalized enrichment score.

In TLE patients, *FABP5*‐expressing astrocytes exhibited upregulated lipid biological processes (Figure 8A). In contrast, astrocytic *Fabp5*‐deficient mice demonstrated downregulated pathways related to lipid metabolism and oxidative stress (Figure 8B). Immunofluorescence staining revealed apparent lipid accumulation in hippocampal astrocytes in KA mice, as indicated by co‐localization of BODIPY 493 with GFAP, which was significantly reduced in *Fabp5*‐deficient mice (Figure 8C–E). Consistently, biochemical quantification showed that *Fabp5* knockout markedly attenuated the increase in triglyceride (TG) levels in the hippocampus following KA induction (Figure 8F). Consistent with the in vivo experiment, *Fabp5* knockdown reduced intracellular TG levels and the mean fluorescence intensity of BODIPY, as assessed by flow cytometry in primary astrocytes in vitro, indicating decreased lipid burden (Figure 8G–I). Given the close association between lipid overload and mitochondrial dysfunction, we next evaluated mitochondrial reactive oxygen species (mtROS) production using MitoSOX and observed a significant reduction in astrocytes with *Fabp5* knockdown. (Figure 8J). Furthermore, JC‐1 staining revealed that *Fabp5* knockdown alleviated mitochondrial membrane potential (ΔΨm) loss, as indicated by a decreased JC‐1 green/red fluorescence ratio (Figure 8K,L). Collectively, these results demonstrate that FABP5 promotes lipid overload and mitochondrial dysfunction in astrocytes under epileptic conditions, providing mechanistic insight into its role in activating downstream cGAS‐STING signaling.

### 2.10 Inhibition of Mitochondrial Fatty Acid Flux Attenuated FABP5‐Induced Mitochondrial Stress, cGAS‐STING Activation, and Downstream Pyroptotic Responses

**FIGURE 9:**
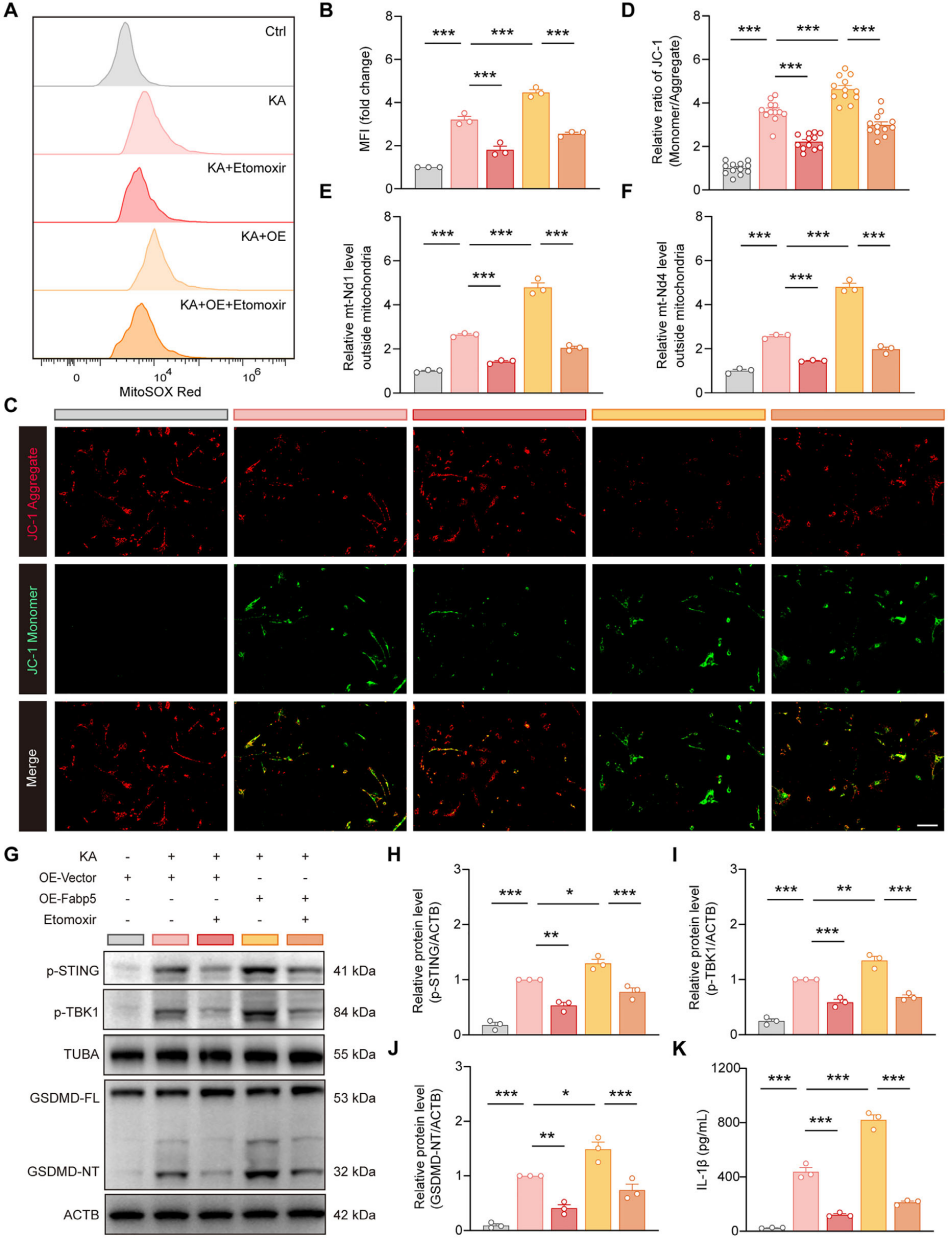
CPT1 inhibition attenuates FABP5‐induced mitochondrial dysfunction and downstream cGAS‐STING‐mediated pyroptotic signaling in astrocytes. (A, B) Flow cytometric analysis (A) and quantification (B) of mtROS levels in primary astrocytes under the indicated conditions (*n* = 3 independent experiments). (C, D) Representative JC‐1 staining images (C) and quantitative analysis (D) of JC‐1 green/red fluorescence ratio (*n* = 12 views from three independent experiments; scale bar, 50 µm). (E, F) Quantification of cytosolic mtDNA levels assessed by qPCR using mitochondrial genes *Nd1* and *Nd4* (*n* = 3 independent experiments). (G–J) Representative immunoblots (G) with quantification of p‐STING (H), p‐TBK1 (I), and GSDMD‐NT (J) protein levels (*n* = 3 independent experiments). (K) The protein level of IL‐1β detected by ELISA in the cell supernatants from primary astrocytes (*n* = 3 independent experiments). Data are represented as means ± SEM. Statistical analysis was performed using one‐way ANOVA followed by Tukey's post hoc tests. ^*^ *p* < 0.05, ^**^ *p* < 0.01, and ^***^ *p* < 0.001. KA, kainic acid; MFI, mean fluorescence intensity.

To further determine whether mitochondrial lipid burden contributes to FABP5‐mediated signaling, we pharmacologically inhibited carnitine palmitoyltransferase 1 (CPT1), a key enzyme regulating mitochondrial fatty acid import, using etomoxir. CM‐KA treatment markedly induced mitochondrial dysfunction in astrocytes, as evidenced by elevated mtROS production (Figure 9A,B), loss of mitochondrial membrane potential (Figure 9C,D), and increased cytosolic mtDNA levels (Figure 9E,F). Importantly, CPT1 inhibition significantly mitigated these alterations. Etomoxir treatment reduced mtROS accumulation, restored mitochondrial membrane potential, and decreased the release of mtDNA into the cytosol compared with the KA group (Figure 9A–F), indicating that limiting mitochondrial fatty acid influx alleviates mitochondrial stress under epileptic conditions. Consistent with these findings, CPT1 inhibition suppressed activation of the cGAS‐STING pathway, as reflected by reduced phosphorylation of STING and TBK1 (Figure 9G–I), and further diminished downstream pyroptotic signaling, including GSDMD cleavage and IL‐1β secretion (Figure 9J,K). Notably, *Fabp5* overexpression further exacerbated CM‐KA‐induced mitochondrial dysfunction, accompanied by augmented cGAS‐STING activation and pyroptotic responses (Figure 9A–K). However, etomoxir treatment effectively reversed these FABP5‐induced effects, reducing mitochondrial stress and downstream signaling to levels comparable to those observed in the KA group, although not to the extent observed in the KA + Etomoxir group. (Figure 9A–K). Collectively, these findings demonstrated that FABP5 promotes mitochondrial dysfunction and subsequent activation of the cGAS–STING pathway at least in part by enhancing mitochondrial lipid burden. These results provide functional evidence supporting a model in which excessive lipid flux to mitochondria serves as an important upstream mechanism linking FABP5 to cGAS‐STING activation and pyroptosis under epileptic conditions.

## 3 Discussion

Our study identified a previously unexplored function of astrocytic FABP5 in the pathogenesis of TLE. FABP5 was increased selectively in astrocytes within the hippocampus of epileptic mice. Importantly, astrocytic *Fabp5* deficiency attenuated seizure activity and neuronal loss by inhibiting astrocytic pyroptosis and neuroinflammation. This finding is intriguing, given that it contradicts the previously reported therapeutic effects of FABP5 in lung inflammation and asthma [28, 29, 46]. As noted in the Introduction, the role of FABP5 in inflammatory diseases remains controversial, and our findings add substantial weight to the evidence supporting its pro‐inflammatory role. The underlying mechanisms by which FABP5 regulates inflammation may vary across different disease contexts and different cell types, thereby leading to divergent effects.

GSDMD‐dependent pyroptosis accounts for the release of inflammatory cytokines in various diseases, whereas a recent study has depicted the role of macrophage GSDMD in tissue regeneration [47], indicating its dual effect under distinct pathologic conditions. Pyroptosis is strongly associated with refractory epilepsy. Our results reveal that inhibition of pyroptosis by *Fabp5* ablation in astrocytes improves the pathological and behavioral phenotypes of epilepsy. Consistently, a recent study demonstrated that suppressing NLRP3 inflammasome‐dependent pyroptosis in neural cells exerted an antiepileptic effect [11]. However, in microglia, which contribute to neuroinflammation in various neurological diseases, insufficient pyroptosis was found to attenuate their phagocytic activity, thereby exacerbating seizure susceptibility and the accumulation of apoptotic neurons in epileptic mice [48]. Therefore, cell‐specific approaches are needed to manipulate pyroptosis to treat TLE as overall inhibition may lead to side effects from impacts on microglia. FABP5 may be a potential molecular target for astrocytic pyroptosis due to its selective expression pattern in TLE. Y18 compound is a dual FABP4/5 inhibitor exhibiting potent inhibitory activity and high selectivity with an excellent safety profile. Oral administration has exhibited significant anti‐inflammatory effects in LPS‐induced liver injury [49]. Meanwhile, several investigations have provided tenable blood‐brain‐barrier‐crossing nanoparticles and modular nano‐platform for drug delivery to the central nervous system targeting astrocytes [50, 51, 52]. Together, these findings make it possible to inhibit FABP5 in astrocytes specifically and to inhibit pyroptosis for epilepsy treatment.

Cyclic GMP‐AMP synthase (cGAS) functions as a cytosolic DNA sensor that generates 2’3’‐cGAMP, thereby activating the adaptor protein stimulator of interferon genes (STING). The cGAS‐STING pathway can detect foreign DNA and tumor‐derived DNA to mediate innate immune defense and intrinsic antitumor immunity [53]. In spite of its protective roles, however, aberrant activation of the cGAS‐STING pathway by self‐DNA has emerged as an important mechanism fueling the development of inflammation [54]. In agreement, our results demonstrate dramatic activation of cGAS‐STING signal in epilepsy, and its downregulation by *Fabp5* knockdown significantly ameliorates neuroinflammation. Mitochondria are highly susceptible to oxidative damage, and their genome is not protected by as robust mechanisms as is the genomic DNA [55]. Therefore, mtDNA is the most common self‐DNA to trigger cGAS activation in the inflammatory process [56]. In the present study, *Fabp5* knockdown noticeably reduced mitochondrial injury and the release of mtDNA into the cytosol, suggesting that mitochondrial dysfunction may serve as an intermediate process connecting FABP5 activity to cGAS‐STING activation

Disruption in lipid metabolism and signaling in astrocytes can lead to pathogenic mechanisms associated with a variety of neurological disorders [57]. Under physiological conditions, astrocytes maintain brain lipid homeostasis partly through storing excess lipids in lipid droplets and coordinating fatty acid trafficking and oxidation [58]. Neuron‐derived fatty acids can be transferred to astrocytes and subsequently utilized through mitochondrial β‐oxidation in response to neuronal activity [59]. Indeed, neuron‐astrocyte metabolic coupling has been shown to protect against activity‐induced fatty acid toxicity by enabling astrocytes to buffer excess neuronal lipids and prevent lipotoxic damage [59]. However, in epilepsy, hyper‐synchronized activity of neurons may trigger excessive fatty acids release, which overwhelms the metabolic capacity of astrocytes, leading to cellular dysfunction and neurotoxicity. In this context, astrocytes may undergo a functional shift from protective lipid buffering to a maladaptive lipid‐handling state characterized by lipid accumulation. These lipid‐laden reactive astrocytes can exacerbate neuronal oxidative stress, as well as activate microglia through IL‐3 signaling to worsen neuroinflammation. Consistently, reactive astrocytes have been shown to induce neuronal death through the production of saturated lipids [60], highlighting the dual role of astrocytes in lipid metabolism under physiological and pathological conditions. In the present study, our data indicate that astrocytic FABP5 plays an important role in regulating intracellular lipid handling under epileptic stress. FABP5 appears to modulate the trafficking and repartitioning of fatty acids within astrocytes. Mechanistically, enhanced FABP5‐dependent intracellular lipid flux may promote the delivery of fatty acids to both lipid storage compartments and mitochondrial oxidative pathways. When mitochondrial processing capacity becomes limiting, excessive lipid influx induces mitochondrial dysfunction, as reflected by increased oxidative stress, membrane depolarization, and structural damage. This, in turn, promotes the release of mtDNA into the cytosol, thereby facilitating activation of the cGAS‐STING pathway and downstream inflammatory and pyroptotic responses. Consistently, in our study, *Fabp5* deficiency attenuated lipid accumulation, reduced mitochondrial injury, decreased cytosolic mtDNA release, and suppressed cGAS‐STING‐mediated pyroptotic signaling. Previous studies have also associated FABP5 with abnormal intracellular lipid accumulation and aggravated pathogenesis [61, 62, 63], further supporting its role in metabolic regulation. Furthermore, there is emerging evidence that lipid overload can drive the transition from adaptive lipid utilization to maladaptive lipotoxicity, ultimately leading to mitochondrial dysfunction [64, 65]. In turn, mitochondria dysfunction may alter lipidome profile, reprogram fatty acid metabolism, and further induce lipid droplet accumulation, forming a feed‐forward loop [66]. Our findings are consistent with this framework and suggest that FABP5 may contribute to this pathological cycle under epileptic conditions. Importantly, pharmacological inhibition of mitochondrial fatty acid import using a CPT1 inhibitor attenuated mitochondrial stress, reduced mtDNA release, and suppressed cGAS‐STING activation, including under conditions of *Fabp5* overexpression. These findings support a functional link between mitochondrial lipid burden and inflammatory signaling, and further indicate that enhanced mitochondrial lipid flux constitutes an important upstream mechanism linking FABP5 to cGAS‐STING activation. However, detailed alterations in lipidome profile after *Fabp5* ablation and whether FABP5 may regulate mitochondria function and the cGAS‐STING signaling through additional mechanisms warrant further investigations.

Astrocytic FABP5 has been reported to mediate endocannabinoid (eCB) transport and regulate synaptic signaling [30, 67, 68]. Given that eCB signaling is frequently associated with neuroprotective and anti‐seizure effects, this may seem inconsistent with our findings suggesting a detrimental role of FABP5 in epilepsy. However, the functional outcomes of eCB signaling in epilepsy are increasingly recognized as context‐dependent [69]. Its effects vary across experimental models, intervention paradigms, ligand concentrations, and receptor targets, with both anticonvulsant and pro‐convulsant outcomes reported [70, 71]. Moreover, as a lipid chaperone, FABP5 is involved in the trafficking of a broad range of bioactive lipids, and its functional outcomes are likely determined by the balance of these pathways in a given pathological context. In our model, the beneficial effects observed upon FABP5 deletion indicate that its pro‐inflammatory and mitochondrial stress‐related functions may outweigh its potential neuroprotective roles mediated through eCB signaling. Taken together, these observations highlight the multifaceted roles of FABP5 in lipid signaling and provide a potential additional mechanism underlying its role in epileptic pathology.

Notably, neuronal loss in our model exhibited a region‐specific pattern, with pronounced vulnerability in the CA1 and CA3 regions, whereas dentate granule cells were relatively preserved. This observation is consistent with the well‐established selective vulnerability of hippocampal subregions in both experimental epilepsy models and human TLE specimens [72, 73]. Previous studies suggest that multiple factors may contribute to the relative resistance of DG neurons, including differences in intrinsic excitability, synaptic connectivity, calcium buffering capacity, and ongoing neurogenesis. Selective vulnerability to kainic acid has been closely linked to the regional distribution of ionotropic glutamate receptors. In particular, kainate receptors (KARs) are highly enriched in CA3 pyramidal neurons, where their activation promotes excitotoxicity, increases reactive oxygen species (ROS), and ultimately leads to neuronal death [74]. In contrast, DG cells exhibit distinct electrophysiological and molecular properties that may confer resistance to excitotoxic injury. In addition, differences in calcium handling may further contribute to regional susceptibility, as DG neurons display reduced Ca^2^^+^ influx under epileptic conditions, whereas CA1 neurons show increased sensitivity to excitotoxic stimuli, partly due to the expression of polyamine‐sensitive NMDA receptors [75, 76]. Together, these findings suggest that the differential neuronal vulnerability observed in our study reflects intrinsic regional properties of the hippocampus and is consistent with the established pattern of hippocampal pathology in epilepsy.

**FIGURE 10:**
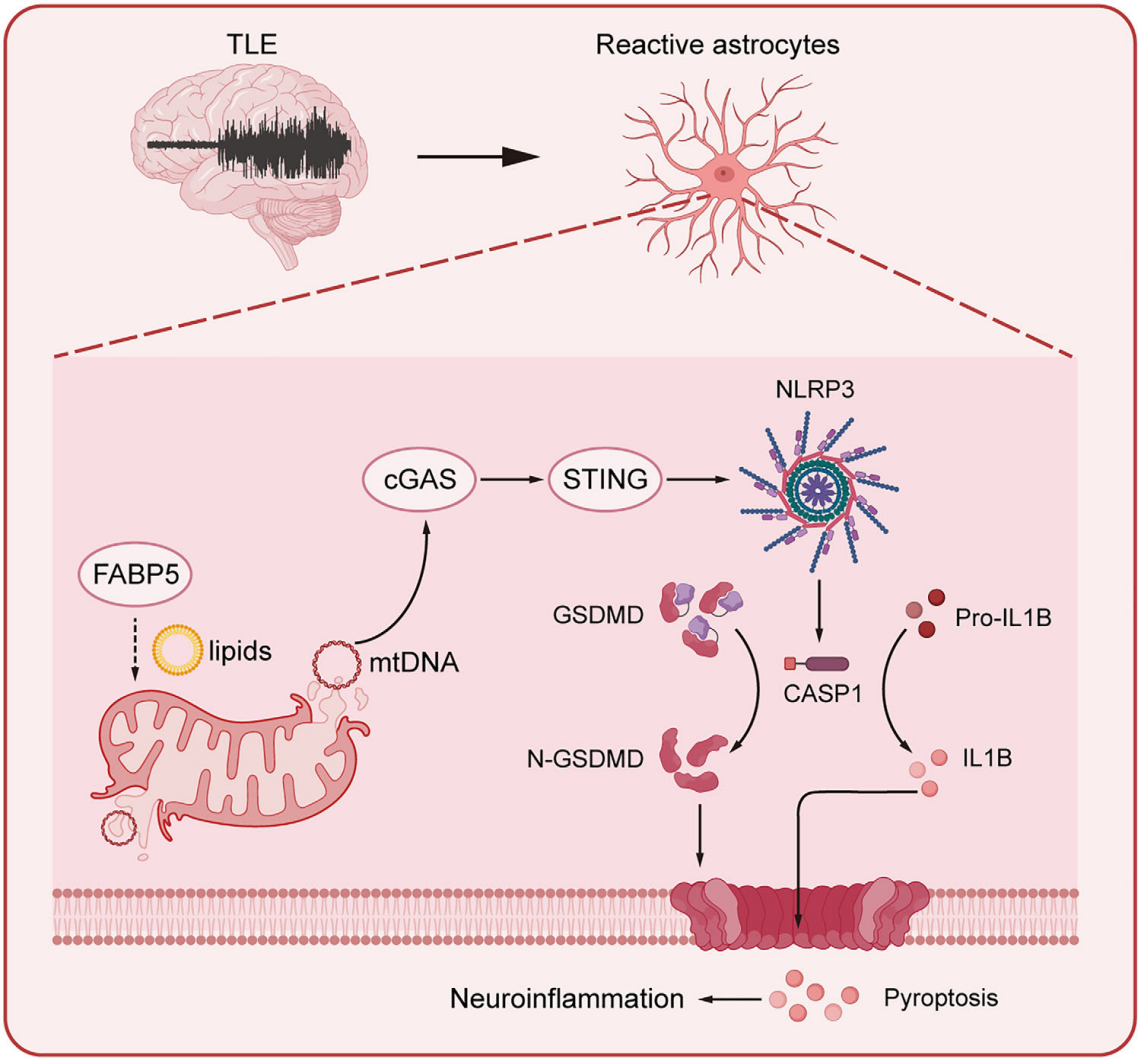
Graphic illustration. Astrocytic FABP5 aggravates pyroptosis and neuroinflammation in epilepsy via mitochondrial lipid burden and the subsequent cGAS‐STING signaling pathway. Created with BioRender.com.

In conclusion, our study demonstrates that astrocytic *Fabp5* deficiency attenuates seizure activity, neuronal loss, and neuroinflammation in TLE by downregulating pyroptosis via cGAS‐STING pathway (Figure 10). Mechanistically, our findings support a model in which astrocytic FABP5 regulates a lipid‐mitochondria‐cGAS‐STING axis. Collectively, these results provide insight into how lipid metabolic stress contributes to neuroinflammation and identify astrocytic FABP5 as a potential disease‐modifying therapeutic target for epilepsy.

## 4 Materials and Methods

### 4.1 Animals

Experimental procedures complied with all the relevant ethical regulations and were approved by the Ethics Committee of Zhongshan Hospital (Approval Number: No.2023‐096). Animal experiments were performed following the ARRIVE guidelines. C57BL/6JGpt‐*Fabp5^em1Cflox^*/Gpt mice and C57BL/6JGpt mice were obtained from GemPharmatech (Shanghai, CHN, stock numbers T009436 and N000013). Adult male mice (8–12 weeks old, 20–25 g) were used in the experiments. Animals were maintained under controlled environmental conditions with a 12 h light/dark cycle, at a temperature of 22°C ± 2°C, with free access to food and water. Mice were housed (4‐5 per cage) throughout the study.

### 4.2 Intraperitoneal Injection of Pilocarpine (Pilo i.p.)

Mice were injected intraperitoneally with atropine (1 mg/kg) 30 min prior to pilocarpine administration. Pilocarpine (350 mg/kg, i.p.) was then administered to induce seizures. If seizures failed to progress to Racine stage IV‐V within 15 min, additional doses of pilocarpine (100 mg/kg, i.p.) were given at intervals until persistent seizures were induced, with no more than three additional injections. Acute convulsive seizures were evaluated using the Racine scale as follows: stage I, mouth and facial movements; stage II, head nodding; stage III, forelimb clonus; stage IV, rearing; stage V, continuous rearing, jumping, and loss of posture. Mice reaching stage IV‐V were included in the subsequent experiments, and seizure was terminated 3 h after induction by intraperitoneal administration of diazepam (0.1%, 10 mg/kg).

### 4.3 Intraperitoneal Injection of Kainic Acid (KA i.p.)

Mice were administered KA (24 mg/kg, K0250, Sigma–Aldrich, St. Louis, MO, USA) via intraperitoneal injection. Acute convulsive seizures were evaluated using the Racine scale as described above. The progression of seizure stages in mice and the latency to the first stage V seizures (if reached) were recorded. Mice reaching stage IV‐V were included in the subsequent experiments, and seizure was terminated 3 h after induction by intraperitoneal administration of diazepam (0.1%, 10 mg/kg).

### 4.4 Intraperitoneal Injection of C‐176 (C‐176 i.p.)

For STING inhibitor treatment, mice were intraperitoneally injected with C‐176 (SML2559, Sigma–Aldrich, USA) at a dose of 750 nmol in 200 µL corn oil every 3 days, starting 1 day after IHKA and continuing for 3 weeks. After drug administration, no obvious abnormalities in behavior or feeding were observed, and the mouse body weight showed no significant differences.

### 4.5 Intrahippocampal Injection of Kainic Acid (IHKA)

Mice were anesthetized with isoflurane delivered in oxygen using a precision vaporizer. Induction was carried out in a closed chamber with 3%–4% isoflurane at a flow rate of 0.5 L/min. Following loss of the toe‐pinch withdrawal reflex, mice were secured in a stereotaxic apparatus (RWD, CHN). 1.5%–2% isoflurane was used for maintenance during the surgical procedure. KA (350 ng in 500 nL) was stereotactically injected into the right hippocampus at the following coordinates relative to bregma (anterioposterior −2.0 mm; mediolateral 1.6 mm; dorsoventral −1.6 mm). The infusion was performed at a rate of 0.1 µL/min using a 5 µL Hamilton syringe mounted on a micro‐syringe pump. The injection needle was kept in place for 5 min before removal to avoid liquid reflux. The incision was then sutured and disinfected with medical iodine. The seizure stage was assessed according to the Racine scale.

### 4.6 Stereotaxic Injection of Virus

Briefly, under isoflurane anesthesia, mice were immobilized on a stereotaxic frame as described above. For bilateral hippocampal delivery, 0.5 µL of adeno‐associated virus was administered at an infusion rate of 0.1 µL/min using coordinates referenced to bregma (anterioposterior −2.0 mm; mediolateral ± 1.6 mm; dorsoventral −1.6/−1.8 mm). AAV‐gfaABC1D‐EGFP‐P2A‐iCre‐WPRE‐pA (S0407‐5) and AAV‐gfaABC1D‐EGFP‐WPRE‐pA (S0246‐5) were purchased from Taitool Bioscience (Shanghai, CHN).

### 4.7 Electrodes Implantation and Electroencephalographic (EEG) Recordings

Mice were anesthetized with isoflurane and placed into the stereotaxic apparatus as described above. After skull exposure, mice were implanted with surface electrodes, including a recording electrode placed over the right hippocampus, a ground electrode placed over the cerebellum, and a reference electrode. Subsequently, the electrodes were secured with dental cement. After a 1 week recovery period, the electrodes were connected to a data acquisition system for continuous electroencephalographic recording over 3 days. During recording, mice were housed individually in recording chambers with free access to food and water and allowed to move freely. Electrographic seizures were identified from EEG recordings using Spike2 software (CED, UK) and were defined as high‐frequency discharges lasting ≥10 s with amplitudes greater than twice the baseline signal [77], using the Spike 2 software (CED, UK).

### 4.8 Transcriptome RNA Sequencing (RNA‐seq)

Transcriptome sequencing was performed by Majorbio Bio‐Pharm Technology (Shanghai, CHN). Total RNA was extracted from the hippocampus of KA and cKOKA mice to construct cDNA libraries and conduct RNA‐seq on the Illumina sequencing platform. Data analysis was performed using R project (R version 4.2.2) and Majorbio Cloud platform. Differentially expressed genes (DEGs) were defined with a cutoff of |fold change| >2 and *p* value < 0.05. The raw sequencing data of mouse hippocampus samples have been deposited in the Genome Sequence Archive in the National Genomics Data Center, China National Center for Bioinformation (GSA: CRA032554).

### 4.9 Quantitative Real‐Time Polymerase Chain Reaction (qRT‐PCR)

Total RNA was isolated from cultured astrocytes or hippocampal tissues with Trizol reagent (15596018, Invitrogen, Carlsbad, CA, USA). Complementary DNA was synthesized using the PrimeScript RT reagent kit (RR037A, Takara, Japan). Quantitative PCR (qPCR) was performed in duplicate using the UltraSYBR Mixture kit (CW0957M, Cwbio, China) on an ABI 7500 Real‐Time PCR system (Applied Biosystems, Waltham, MA, USA). Relative gene expression levels were calculated using the 2^−ΔΔCt^ method [78]. For measurement of cytosolic mtDNA, astrocytes were lysed in a digitonin‐based buffer (150 mm NaCl, 50 mm HEPES, 25 µg/mL digitonin, pH 7.4). After incubation at room temperature for 10 min, samples were centrifuged at 17 000 × g for 20 min, and the resulting supernatant containing cytosolic fractions was collected. DNA was extracted using a DNA isolation kit (DC102‐01, Vazyme, CHN) according to the manufacturer's instructions for qPCR. The copy number of mtDNA obtained from cytosolic extracts was normalized to the copy number of nuclear DNA (Tert) obtained from the whole‐cell extracts. Primer sequences are listed in Table 1.

### 4.10 Enzyme‐Linked Immunosorbent Assay (ELISA)

Hippocampal tissues and astrocyte culture supernatants were collected for analysis. For tissue samples, total protein concentration was determined using a BCA assay kit (P0010, Beyotime, CHN). The level of IL‐1β was quantified using a commercial ELISA kit (MLB00C, R&D Systems, Minneapolis, MN, USA) according to the manufacturer's protocol. All samples were measured in duplicate, and standard curves were generated using SoftMax Pro 7.1 software (Molecular Devices, Shanghai, CHN).

### 4.11 Western Blotting

Hippocampal tissues and cultured astrocytes were collected and lysed in RIPA buffer (P0013B, Beyotime, CHN) supplemented with protease inhibitor PMSF (1:100, ST507, Beyotime, CHN) and phosphatase inhibitors (1:50, P1082, Beyotime, CHN). Protein concentrations were determined using a BCA assay kit (P0010, Beyotime, CHN). Equal amounts of protein were separated by SDS‐PAGE and transferred onto PVDF membranes (IPVH00010, Millipore, Bedford, MA, USA). Membranes were blocked with 5% bovine serum albumin for 2 h at room temperature, followed by incubation with primary antibodies overnight at 4°C. The primary antibodies used were as follows: anti‐FABP5 (12348‐1‐AP, 1:1 000; Proteintech, CHN), anti‐NLRP3 (15101, 1:1 000, Cell Signaling, Danvers, MA, USA), anti‐GSDMD (ab209845, ab219800, 1:1 000, Abcam, UK), anti‐cleaved N‐terminal GSDMD (ab215203, 1:1 000, Abcam, UK), anti‐CASP1 (24232, 1:1 000, Cell Signaling, USA), anti‐CASP1/P20 (22915‐1‐AP, 1:1 000, Proteintech, CHN), anti‐phospho‐TBK1 (Ser172, 5483, 1:1 000, Cell Signaling, USA), anti‐TBK1 (83686‐3‐RR, 1:1 000, Proteintech, CHN), anti‐phospho‐STING (Ser365, 72971, 1:1 000, Cell Signaling, USA), anti‐STING (13647, 1:1 000, Cell Signaling, USA), anti‐β‐actin (AF5001, 1:2 000, Beyotime, CHN) and anti‐α‐tubulin (11224‐1‐AP, 1:5 000, Proteintech, CHN). After washing with TBS‐T (0.05% Tween‐20), membranes were incubated with HRP‐conjugated secondary antibodies for 1 h at room temperature. The second antibodies used were as follows: goat‐anti‐rabbit IgG HRP‐conjugated (7074, 1:2 000, Cell Signaling, USA), horse‐anti‐mouse IgG HRP‐conjugated (7076, 1:2 000, Cell Signaling, USA). Protein bands were visualized using enhanced chemiluminescence reagents (SQ201, Epizyme, CHN). Band densities were quantified using ImageJ software (Rawak Software Inc., Stuttgart, GER).

### 4.12 Immunofluorescence Staining

Following deep anesthesia induced by intraperitoneal sodium pentobarbital, mice underwent transcardial perfusion with ice‐cold PBS and then 4% paraformaldehyde. Mice brains were collected, cryoprotected in sucrose solution, embedded in optimal cutting temperature compound, and sectioned at 30 µm using a cryostat (Leica, Wetzlar, GER), with sections mounted onto glass slides. For in vitro experiments, astrocytes were plated on pre‐coated glass coverslips. After removal of the culture medium, cells were rinsed with PBS and fixed in ice‐cold methanol for 30 min. Tissue sections and cultured cells were then incubated in blocking buffer (P0260, Beyotime, CHN) at room temperature for 1 h, followed by incubation with primary antibodies at 4°C overnight and corresponding fluorescent secondary antibodies at room temperature for 2 h. For lipid staining, BODIPY (10 µm, HY‐W090090, MedChemExpress, CHN) was applied for 30 min after secondary antibody incubation. Slides were then mounted with DAPI Fluoromount‐G (0100‐20, Southern Biotech, AL, USA). The primary antibodies used were as follows: anti‐GFAP (MAB360, 1:1 000, Millipore, USA), anti‐NeuN (MAB377, 1:500, Millipore, USA), anti‐IBA‐1 (019‐19741, 1:500, Wako, JPN), anti‐FABP5 (AF3077, 1:200; R&D, USA), anti‐GSDMD (AF4012, 1:200, Affinity, CHN), anti‐cleaved N‐terminal GSDMD (ab215203, 1:200, Abcam, UK), anti‐cGAS (31659, 1:500, Cell Signaling, USA) and anti‐TOM20 (GB111481, 1:200, Servicebio, CHN). Images were acquired using either a fluorescence microscope (IX53, Olympus, Tokyo, JPN) or a laser scanning confocal microscope (FV3000, Olympus, JPN), with appropriate objectives depending on the sample type. Imaging parameters, including gain and offset, were kept consistent across all groups. For co‐localization analysis, Z‐stack images (1 µm intervals) were collected using a 40 × objective.

Image analysis was carried out using ImageJ software (Rawak Software Inc., Stuttgart, GER). All images were processed using identical threshold settings, and quantitative measurements such as area or mean fluorescence intensity were obtained within defined regions of interest for each animal. Mitochondrial morphology was analyzed using the Mitochondria Analyzer plugin. For neuronal quantification, three coronal sections per mouse at comparable anatomical levels were selected for analysis. Positive neurons were initially quantified using the Analyze Particles function in ImageJ, followed by manual validation and correction to ensure accuracy. The average value per animal was used for statistical analysis. For visualization, image brightness and contrast were adjusted uniformly across the entire image after analysis.

### 4.13 Cell Transfection

Lentivirus package was performed using GeneReal So‐easy Lentivirus Packaging Kit (L002C‐20T, Jingruibio, CHN). Briefly, HEK‐293T cells were transfected with lentiviral plasmid mix and indicated plasmids, including plasmids expressing *Fabp5* or *Sting* (Genechem, Shanghai, CHN) and plasmids containing *Fabp5* shRNA (TRCN0000011895, Sigma–Aldrich, USA), in serum‐free Dulbecco's Modified Eagle Medium (DMEM, U21‐265B, Yobibio, CHN) using PEI reagent for 6 h. Cell medium was then replaced with DMEM containing 10% (v/v) fetal bovine serum (FBS, U11‐059A, Yobibio, CHN) and 100 × titer enhancer (1:100) according to the manufacturer's instructions. The lentiviral supernatant was harvested after 48 h and 72 h, centrifuged, concentrated, and stored at −80°C. The lentivirus was added to primary astrocytes 72 h before further stimulation.

### 4.14 Primary Neuronal Culture

Hippocampal tissues were isolated from postnatal day 1 (P1) C57BL/6J mice and enzymatically dissociated with trypsin (25200‐072, Gibco, CA, USA) at 37°C for 20 min, followed by gentle trituration using a 1 mL pipette to obtain a single‐cell suspension. The digestion was terminated by adding DMEM containing 10% (v/v) FBS, and cells were seeded onto poly‐L‐lysine‐coated T25 flasks. After 4 h, the culture medium was replaced with neurobasal medium (21103‐049, Gibco, USA) supplemented with 2% (v/v) B27 (17504‐044, Gibco, USA) and 1% penicillin–streptomycin (15140‐122, Gibco, USA). To suppress glial proliferation, cytosine β‐D‐arabinofuranoside (Ara‐C, 5 µm; C1768, Sigma–Aldrich, USA) was added at 2 days in vitro (DIV2) and maintained for an additional 2 days. Culture medium was partially replaced with fresh maintenance medium every 4 days, and neurons were used for subsequent experiments after 7–10 days in vitro.

### 4.15 Primary Culture of Astrocytes

Primary astrocytes were isolated from the hippocampi of postnatal day 1 (P1) C57BL/6 mice. After removal of meninges and blood vessels, tissues were enzymatically digested with trypsin (25200‐072, Gibco, USA) at 37°C for 20 min. The resulting cell suspension was passed through a 70 µm cell strainer (431752, Corning, NY, USA) and centrifuged at 1000 rpm for 5 min. Cell pellets were reconstituted in Dulbecco's Modified Eagle Medium/Nutrient Mixture F‐12 (DMEM/F‐12) medium (C11330500BT, Gibco, USA) supplemented with 10% (v/v) FBS and 1% (v/v) penicillin–streptomycin, and cells were then seeded into poly‐L‐lysine‐coated T25 flasks. The medium was refreshed every 3 days. After 12 days in vitro, astrocytes were purified by shaking the cultures at 200 rpm for 4 h to remove non‐astrocytic cells, and the adherent astrocytes were retained for subsequent experiments.

### 4.16 KA‐Induced Cell Culture Model

An in vitro model was generated according to a published protocol [31], with minor modifications. Primary neurons were incubated with or without KA (100 µm) for 12 h, after which neuronal conditioned medium (CM) was collected. Primary astrocytes were treated with CM at 72 h following lentivirus transduction for 6–8 h to measure the mitochondrial damage and the cGAS‐STING signal, and for 8–12 h to measure the pyroptosis and inflammatory cytokines. Where indicated, astrocytes were pretreated with etomoxir (30 µm) for 1 h prior to CM stimulation.

### 4.17 Flow Cytometry for BODIPY 493 and MitoSOX Staining

Primary astrocytes were treated as indicated, washed with PBS, and dissociated into single‐cell suspensions. For lipid detection, cells were incubated with BODIPY 493 (2 µm) for 30 min at 37°C in the dark. For mitochondrial ROS measurement, cells were incubated with MitoSOX Red (5 µm, S0061S, Beyotime, CHN) for 20 min at 37°C in the dark. After staining, cells were washed twice with PBS and resuspended for analysis. Flow cytometry was performed using a CytoFLEX flow cytometer (Beckman Coulter, Brea, CA, USA). BODIPY and MitoSOX signals were detected in the FITC and PE channels, respectively. At least 10,000 events were collected per sample. Data were analyzed using FlowJo software (FlowJo LLC, Ashland, OR, USA), and mean fluorescence intensity (MFI) or the percentage of positive cells was quantified.

### 4.18 JC‐1 Staining

Mitochondrial membrane potential was assessed using a JC‐1 Mitochondrial Membrane Potential Assay Kit (C2003S, Beyotime, CHN) according to the manufacturer's instructions. Primary astrocytes were incubated with JC‐1 working solution at 37°C for 20 min in the dark, washed twice with staining buffer, and immediately imaged using a fluorescence microscope (IX53, Olympus, Tokyo, JPN). Mitochondrial membrane potential was evaluated by calculating the green/red fluorescence intensity ratio using ImageJ software (Rawak Software Inc., Stuttgart, GER). An increased green/red ratio reflects mitochondrial depolarization, while a decreased ratio indicates preserved mitochondrial membrane potential.

### 4.19 Triglyceride Measurement

Triglyceride (TG) levels were measured using the Triglyceride Quantification Kit (MAK564, Sigma–Aldrich, St. Louis, MO, USA) according to the manufacturer's instructions. Primary astrocytes or hippocampal tissues were homogenized in assay buffer on ice, followed by centrifugation (12,000 × g, 10 min, 4°C). Supernatants were used for analysis. TG levels were determined by measuring absorbance at 570 nm using a microplate reader (SpectraMax i3x, Molecular Devices, San Jose, CA, USA).

### 4.20 Statistical Analysis

Quantitative data are presented as mean ± standard error of the mean (SEM) from at least three independent biological replicates. Sample sizes for in vivo experiments were determined based on prior experience with the KA‐induced epilepsy model, variability observed in pilot studies, and ethical considerations to minimize animal use. For in vitro experiments, assays were conducted using at least three biologically independent experiments, in accordance with common practice in mechanistic cell biology studies. Sample sizes were determined based on considerations of experimental reproducibility and technical feasibility. Statistical analyses were conducted using GraphPad Prism 8.0 (GraphPad Software, San Diego, CA, USA). One‐way analysis of variance (ANOVA) with Tukey's post hoc test was used for multiple‐group comparisons. Unpaired Student's *t*‐test was applied for two‐group comparisons, and the Mann‐Whitney *U* test was used for non‐normally distributed data. A *p*‐value < 0.05 was considered statistically significant.

## Author Contributions

C.C, X.W, K.H.Z, and J.D conceived and designed the study. C.C and Y.Z performed the experiments and analyzed the results. C.C wrote the manuscript. C.C, Y.Z, K.H.Z, and J.D revised the manuscript. Y.Y.L, Y.F.H, T.T.W, L.F.G, Y.L.Y, and J.Q.Z participated in the data acquisition, analysis, and interpretation. K.H.Z and J.D provided funding support. All authors read and approved the final manuscript.

## Funding

This work was supported by the grants from the National Natural Science Foundation of China (82271499 to J.D., 82171444 to X.W., 32301013 to K.Z.) and the Shanghai Pujiang Program (22PJ1402100 to K.Z.)

## Ethics Statement

Experimental procedures complied with all the relevant ethical regulations and were approved by the Ethics Committee of Zhongshan Hospital (Approval Number: No.2023‐096).

## Conflicts of Interest

The authors declare no conflicts of interest.

## Supporting information




**Supporting File**: advs76377‐sup‐0001‐SuppMat.docx.

## Data Availability

The data that support the findings of this study are available from the corresponding author upon reasonable request.
