# Supplementary material for: Fatty Acid Binding Protein 5 Mediates Astrocytic Pyroptosis and Neuroinflammation in Epilepsy via cGAS/STING Pathway
Source: Adv Sci (Weinh). 2026 Jul 6:e76377. Online ahead of print. doi: 10.1002/advs.76377 (PMC13335901; doi:10.1002/advs.76377)
Supplement: Supplementary file 1 — Supporting File: advs76377‐sup‐0001‐SuppMat.docx. [file ADVS-9999-e76377-s001.docx]

**SUPPLEMENTARY INFORMATION**

**Fatty acid binding protein 5 mediates astrocytic pyroptosis and neuroinflammation in epilepsy *via* cGAS/STING pathway**

Chen Chen, Yao Zhao, Yangye Lian, Yifei Hou, Lifen Gong, Tingting Wu, Yulin Yang, Jiaqi Zhu, Xin Wang, Kaihua Zhang, Jing Ding


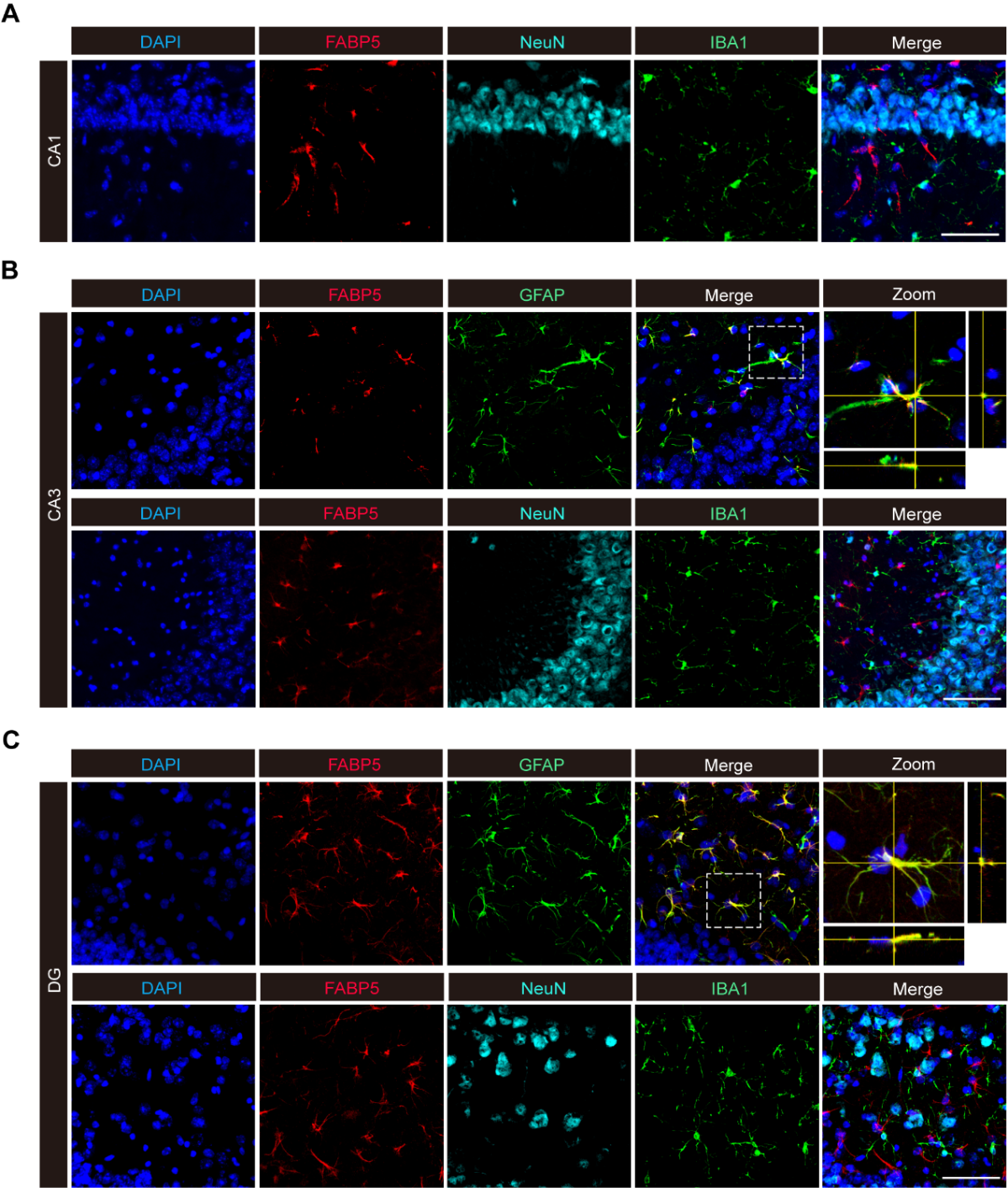


**Figure S1 FABP5 is mainly expressed in astrocytes in the hippocampus.** **(A-C)** Representative immunofluorescent images of FABP5 (red) and GFAP (green) double labeling and images of FABP5 (red), NeuN (cyan) and IBA1 (green) triple labeling in the CA1 (A), CA3 (B) and DG (C) region within the hippocampus (scale bar, 50 μm).

**
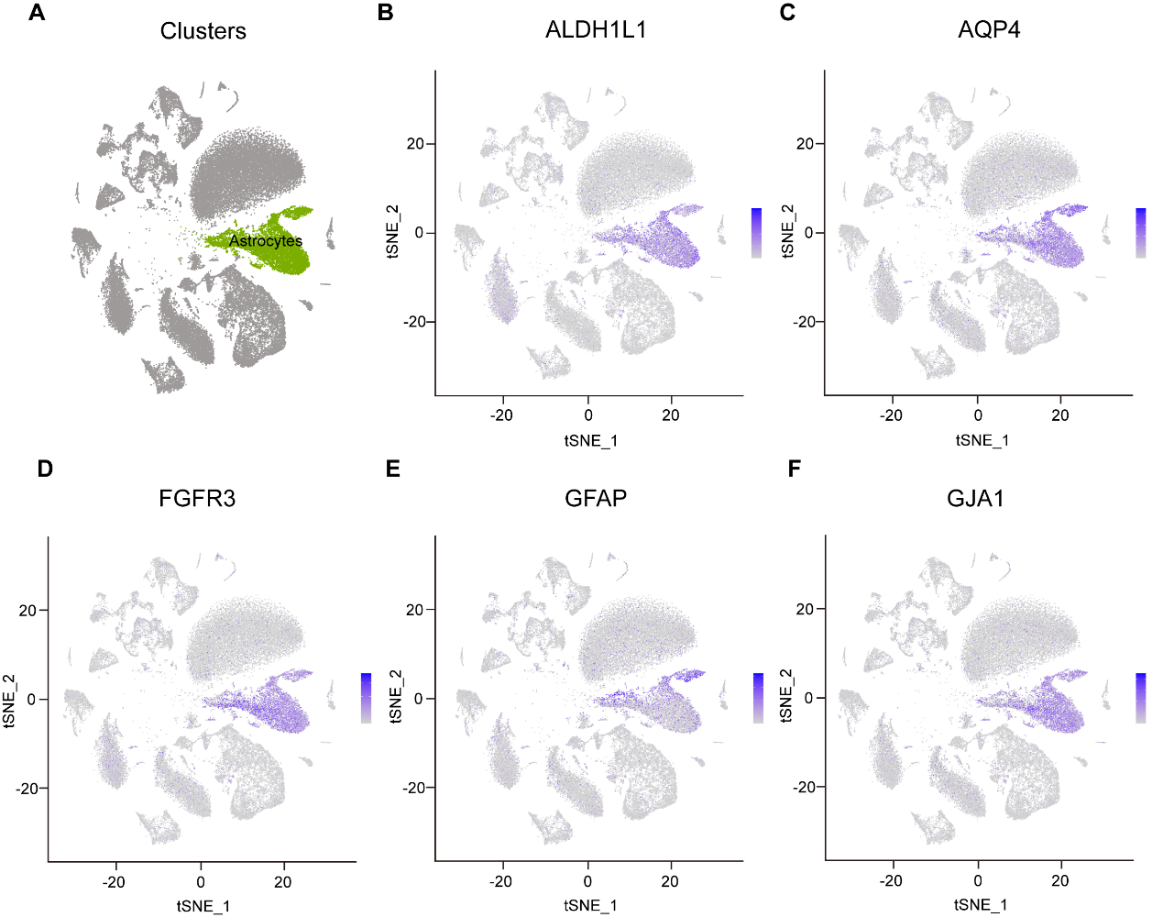
**

**Figure S2 Definition of astrocytes in single-nucleus RNA sequencing data. (A)** Unsupervised clustering of cell populations with green-coded astrocytes. **(B-F)** Expression scatterplots of astrocyte markers in cell populations from (A). Single-nucleus RNA sequencing data are available from NCBI GEO database under accession number GSE190452.


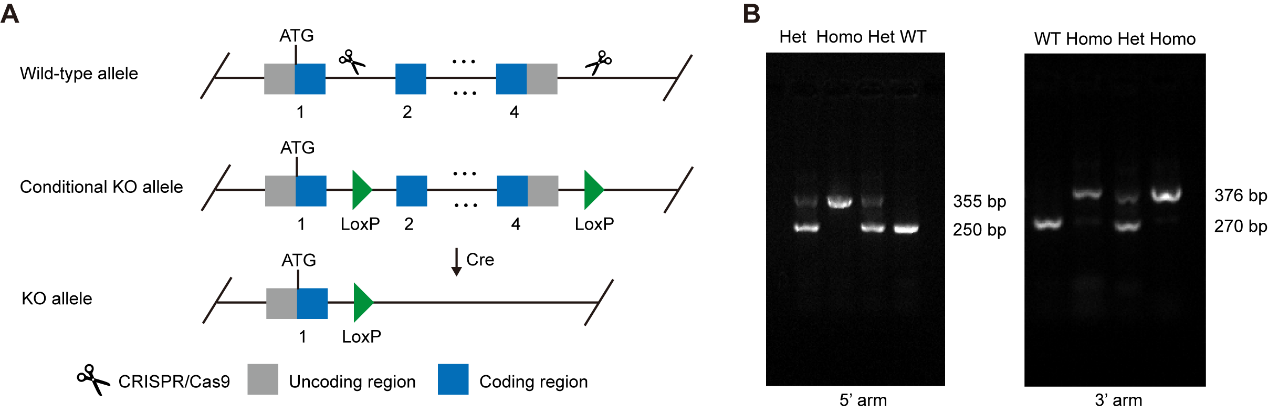


**Figure S3 Validation of *Fabp5*^fl/fl^ transgenic mice. (A)** Schematic illustration of the CRISPR/Cas9-mediated generation of transgenic mice, with loxP sites flanking the target exon, and the subsequent Cre-mediated excision leading to *Fabp5* knockout. **(B)** PCR-based genotyping analysis of transgenic mice, demonstrating the expected band patterns corresponding to wild-type (WT), heterozygous (Het), and homozygous (Homo) genotypes.


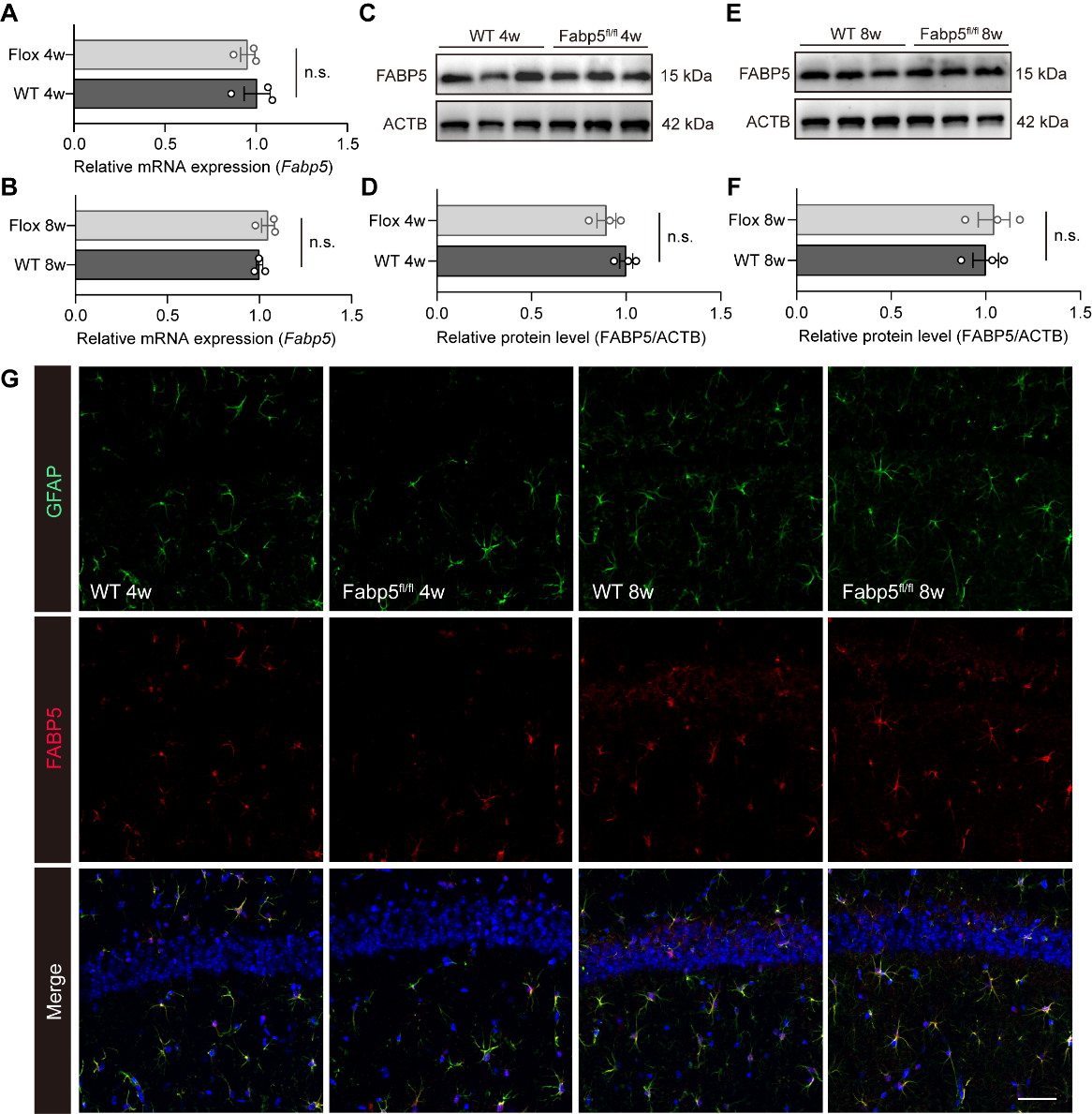


**Figure S4 Baseline phenotypic characterization of *Fabp5*^fl/fl^ mice. (A-B)** The mRNA expression levels of *Fabp5* in the hippocampus of 4- and 8-week-old mice (n = 3 mice). **(C-F)** Representative immunoblots with statistical analysis of FABP5 protein levels in the hippocampus of 4- and 8-week-old mice (n = 3 mice). **(G)** Representative immunofluorescent images of GFAP (green) and FABP5 (red) double labeling in the hippocampus (scale bar, 50 μm). Data are represented as means ± SEM. Statistical analysis was performed using two-sided unpaired Student’s t-tests. n.s., not significant.


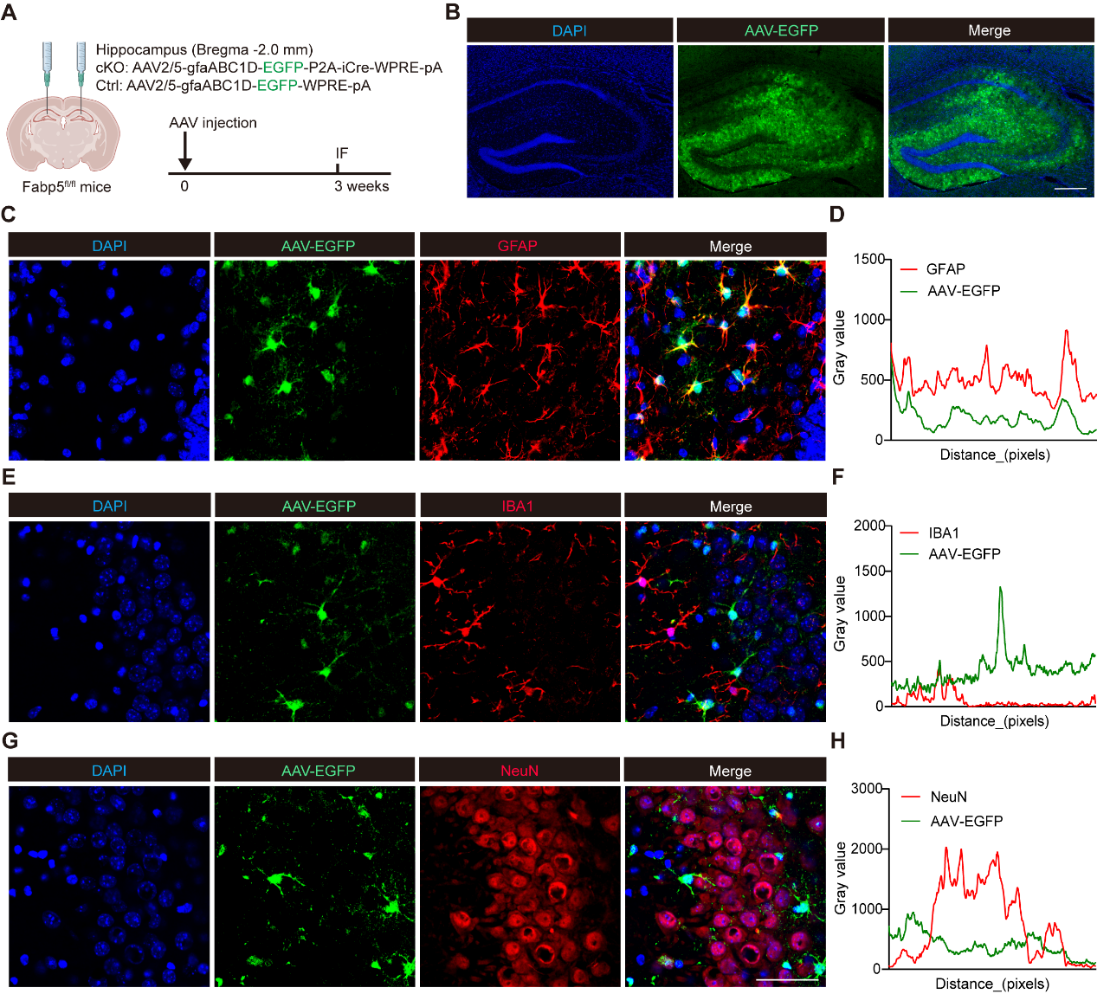


**Figure S5 Validation of AAV transduction and cell-type specificity. (A)** Schematic of virus injection targeting astrocytes into the hippocampus of *Fabp5*^fl/fl^ mice. **(B)** Representative immunofluorescent images of AAV-gfaABC1D-EGFP-iCRE expression in the hippocampus (scale bar, 500 μm). **(C-D)** Representative immunofluorescent images (C) and signal quantification (D) of GFAP (red) and AAV double labeling in the hippocampus (scale bar, 50 μm). **(E-F)** Representative immunofluorescent images (E) and signal quantification (F) of IBA1 (red) and AAV double labeling in the hippocampus (scale bar, 50 μm). **(G-H)** Representative immunofluorescent images (G) and signal quantification (H) of NeuN (red) and AAV double labeling in the hippocampus (scale bar, 50 μm).


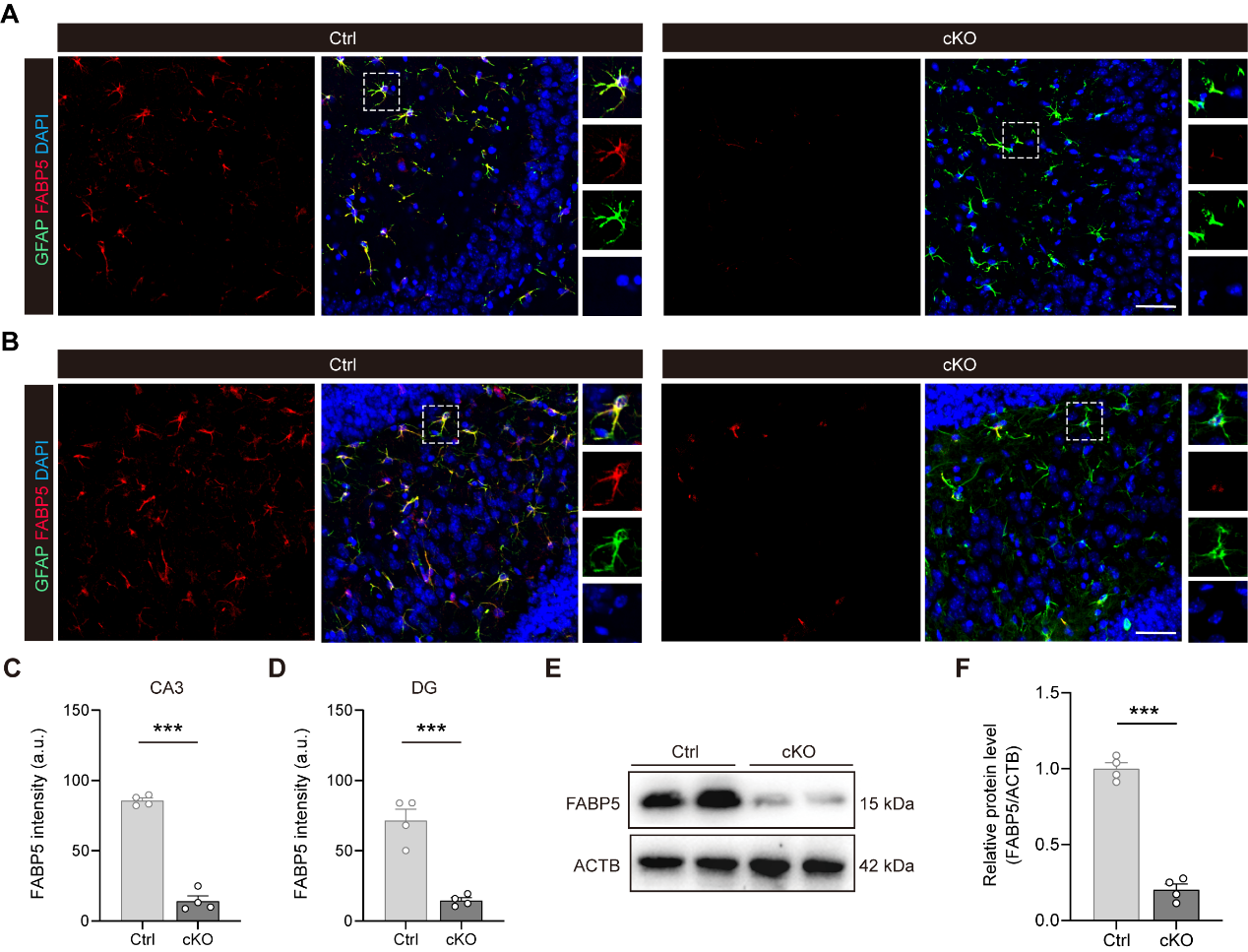


**Figure S6 *Fabp5* is specifically knocked out in astrocytes.** **(A-B)** Representative immunofluorescent images of GFAP (green) and FABP5 (red) double labeling in the hippocampal CA3 (A) and DG (B) regions (scale bar, 50 μm). **(C-D)** Quantification of FABP5 intensity in GFAP+ astrocytes in the CA3 (C) and DG (D) regions within the hippocampus after AAV injection (n = 4 mice). (**E-F**) Representative immunoblots (E) and statistical analysis (F) of FABP5 protein levels in the hippocampus (n = 4 mice). Data are represented as means ± SEM. Statistical analysis was performed using two-sided unpaired Student’s t-tests. ^***^*p* < 0.001. cKO, conditional knockout. a.u., arbitrary units.


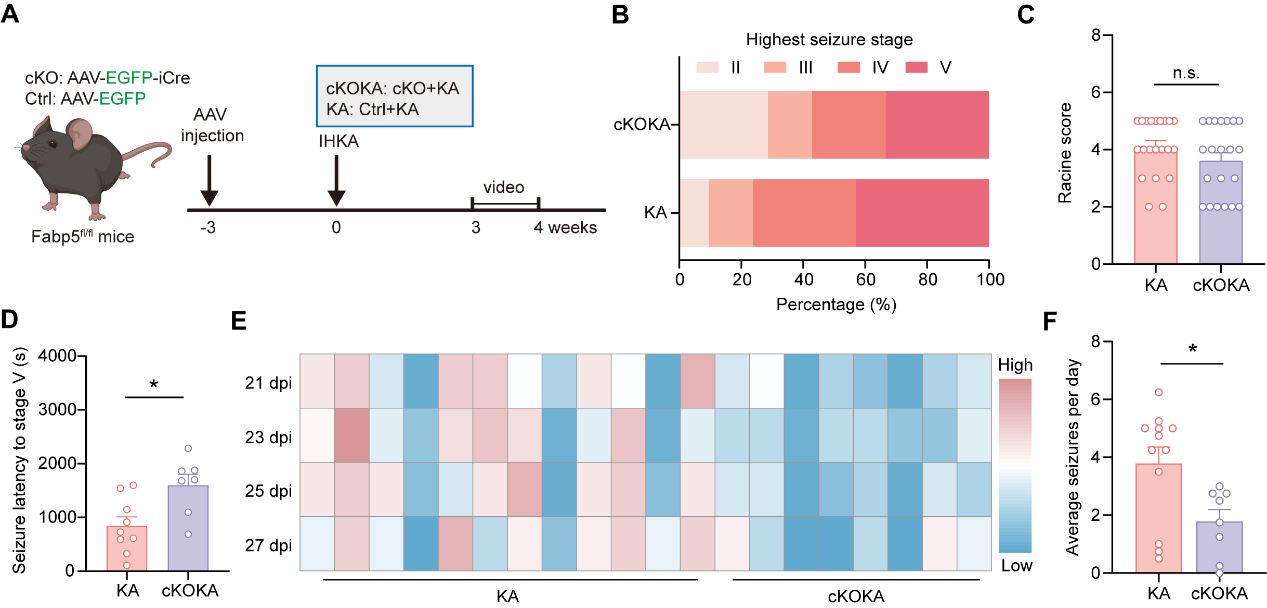


**Figure S7 Conditional knockout of *Fabp5* in astrocytes prior to IHKA reduces seizures** **susceptibility and decreases** **spontaneous recurrent seizures. (A)** Graphic illustration of the experimental timeline for *Fabp5*^fl/fl^ mice injected with AAV prior to IHKA. **(B-C)** Distribution of the highest seizure stage reached according to Racine scores in mice following IHKA (n = 21 mice). **(D)** Quantification of latency to the first generalized seizure (KA, n = 9; cKOKA, n = 7 mice). **(E-F)** Heatmap and quantification of seizure events during 3-4 weeks after IHKA (KA, n = 12; cKOKA, n = 8 mice). Data are represented as means ± SEM. Statistical analysis was performed using Mann-Whitney test (F) and two-sided unpaired Student’s t-tests (C, D). ^*^*p* < 0.05. n.s., not significant; cKO, conditional knockout.


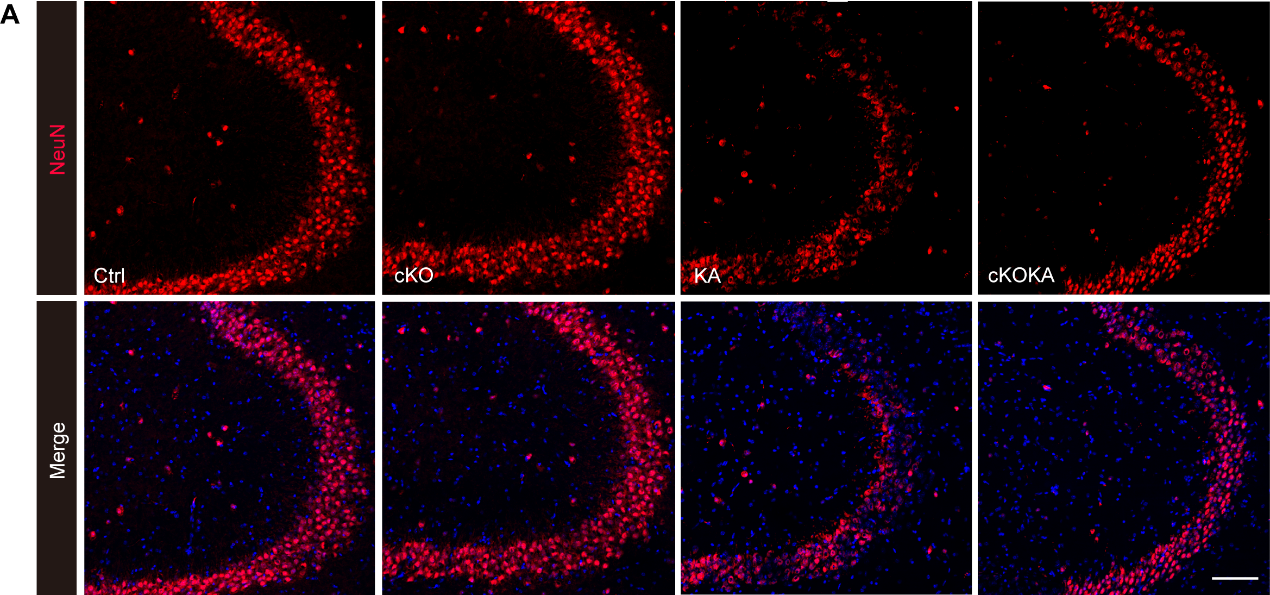


**Figure S8 Conditional knockout of *Fabp5* in astrocytes attenuates neuronal loss in the hippocampal CA3 in epileptic mice.** **(A)** Representative immunofluorescent images of NeuN (red) labeling in the hippocampus (scale bar, 100 μm). cKO, conditional knockout.


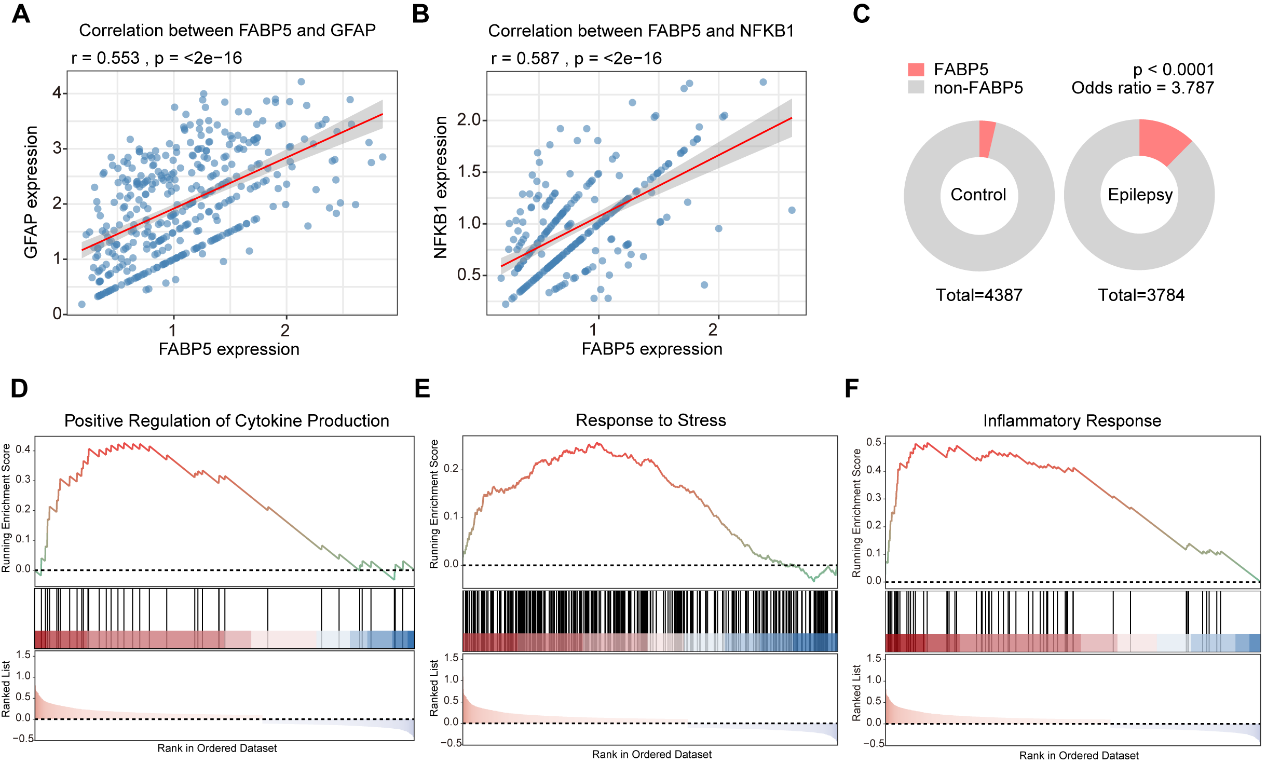


**Figure S9 *FABP5* expression is related to reactive activation of astrocytes and inflammation. (A)** Correlation of the mRNA expression levels between *GFAP* and *FABP5* in the astrocytes from TLE patients. **(B)** Correlation of the mRNA expression levels between *NFKB1* and *FABP5* in the astrocytes from TLE patients. **(C)** Ratio of *FABP5*-expressing astrocytes in total astrocytes from controls and TLE patients. **(D-F)** Representative upregulated pathways related with inflammation in *FABP5*-expressing astrocytes compared to *FABP5* non-expressing astrocytes from TLE patients.


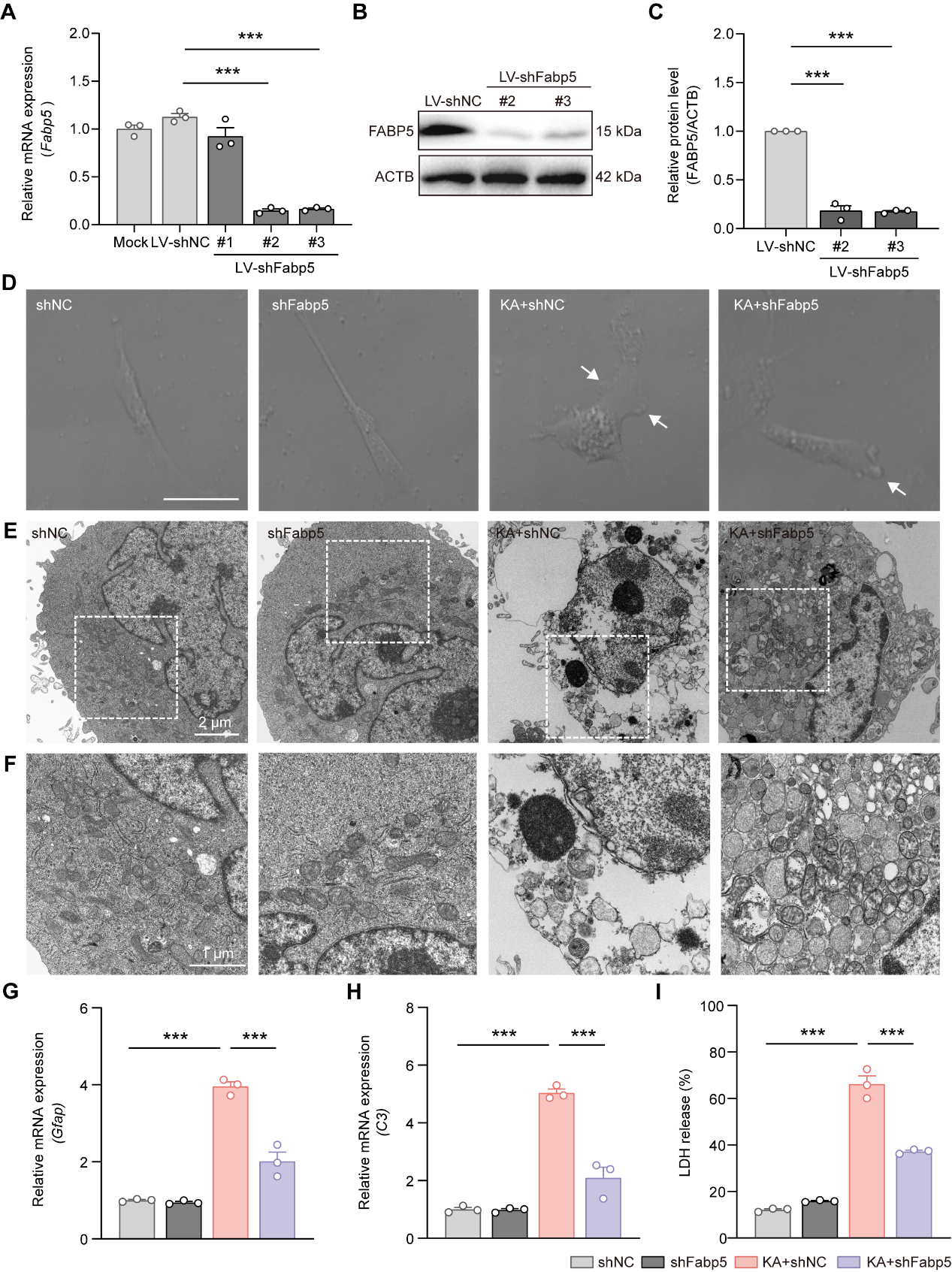


**Figure S10 *Fabp5* knockdown attenuates pyroptosis in primary astrocytes. (A)** The mRNA expression level of *Fabp5* in primary astrocytes transfected with *Fabp5*-shRNA lentivirus or negative lentivirus (n = 3 independent experiments). **(B-C)** Representative immunoblots (B) with statistical analysis (C) of FABP5 protein levels in primary astrocytes transfected with *Fabp5*-shRNA lentivirus or negative lentivirus (n = 3 independent experiments). **(D)** Representative images showing morphological alterations of primary astrocytes in each group (scale bar, 50 μm). **(E-F)** Representative transmission electron microscopy images showing ultrastructural features in each group (E) and higher-magnification images of the regions indicated by white boxes (F). **(G-H)** The mRNA expression levels of *Gfap* (G) and *C3* (H) in CM-KA-primed primary astrocytes transfected with *Fabp5*-shRNA lentivirus or negative lentivirus (n = 3 independent experiments). **(I)** Levels of LDH release in the cell supernatants from primary astrocytes (n = 3 independent experiments). Data are represented as means ± SEM. Statistical analysis was performed using one-way ANOVA followed by Tukey’s post hoc tests. ^***^*p* < 0.001. KA, kainic acid; NC, negative control.


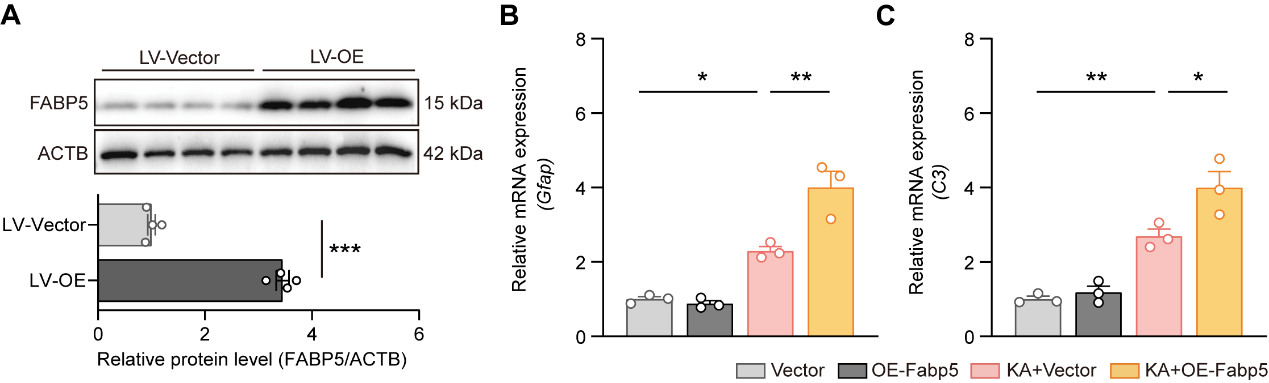


**Figure S11 *Fabp5* overexpression aggravates pyroptosis in primary astrocytes. (A)** Representative immunoblots with statistical analysis of FABP5 protein levels in primary astrocytes transfected with *Fabp5*-overexpressing lentivirus or vector (n = 4 independent experiments). **(B-C)** The mRNA expression levels of *Gfap* (B) and *C3* (C) in CM-KA-primed primary astrocytes transfected with *Fabp5*-overexpressing lentivirus or vector (n = 3 independent experiments). Data are represented as means ± SEM. Statistical analysis was performed using two-sided unpaired Student’s t-tests (A) and one-way ANOVA followed by Tukey’s post hoc tests (B, C). ^*^*p* < 0.05, ^**^*p* < 0.01, and ^***^*p* < 0.001. OE, overexpression.


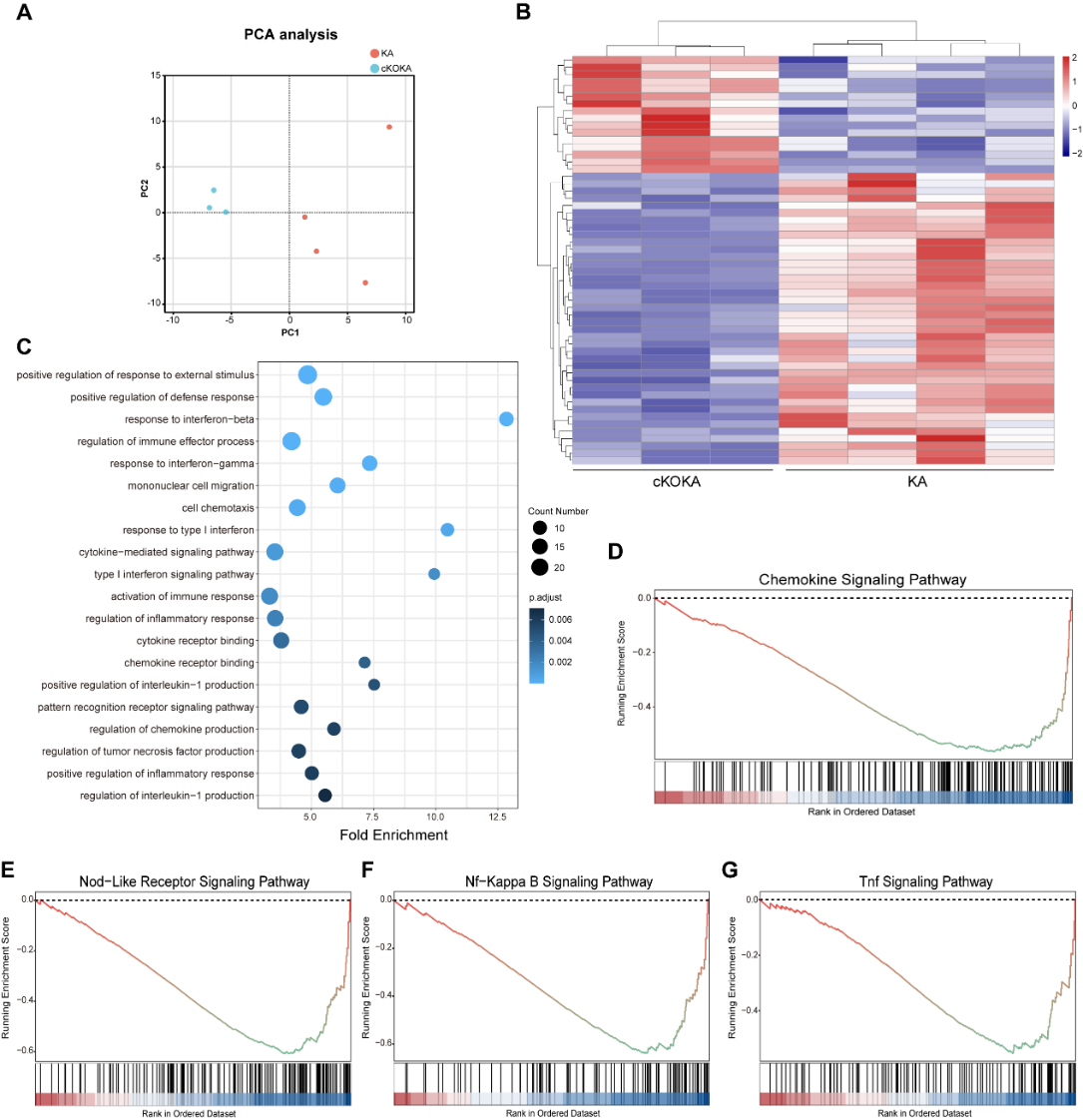


**Figure S12 Conditional knockout of *Fabp5* in astrocytes downregulates pyroptosis- and inflammation-related pathway. (A-B)** PCA plot (A) and heatmap (B) showing the expression of top variant genes in the hippocampus from cKOKA mice and KA mice. **(C)** Gene ontology (GO) analysis of identified DEGs showing the top enriched biological processes between cKOKA mice and KA mice. A cutoff with |fold change| >2 and p value < 0.05 was used to define DEGs. **(D-G)** Gene set enrichment analysis (GSEA) showing the down-regulated pathways regarding pyroptosis and neuroinflammation in the hippocampus from cKOKA mice compared to KA mice.


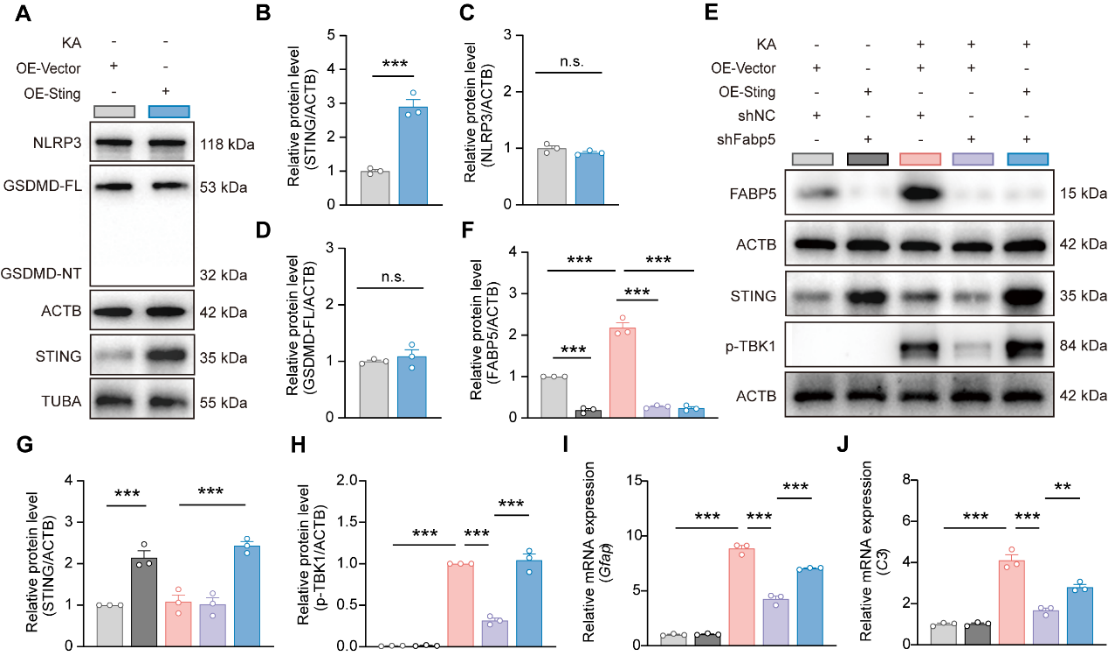


**Figure S13** ***Sting* overexpression partially counteracts the reduction in pyroptosis by *Fabp5* knockdown. (A-D)** Representative immunoblots (A) with statistical analysis (B-D) of STING (B), NLRP3 (C) and GSDMD-FL (D) protein levels in primary astrocytes transfected with *Sting*-overexpressing lentivirus or vector (n = 3 independent experiments). **(E-H)** Representative immunoblots (E) with statistical analysis (F-H) of FABP5 (F), STING (G) and p-TBK1 (H) protein levels in CM-KA-primed *Fabp5*-knockdown primary astrocytes transfected with *Sting*-overexpressing lentivirus or vector (n = 3 independent experiments). **(I-J)** The mRNA expression levels of *Gfap* (I) and *C3* (J) in CM-KA-primed *Fabp5*-knockdown primary astrocytes transfected with *Sting*-overexpressing lentivirus or vector (n = 3 independent experiments). Data are represented as means ± SEM. Statistical analysis was performed using two-sided unpaired Student’s t-tests (B-D) and one-way ANOVA followed by Tukey’s post hoc tests (F-J). ^**^*p* < 0.01, and ^***^*p* < 0.001. KA, kainic acid; NC, negative control; n.s., not significant.


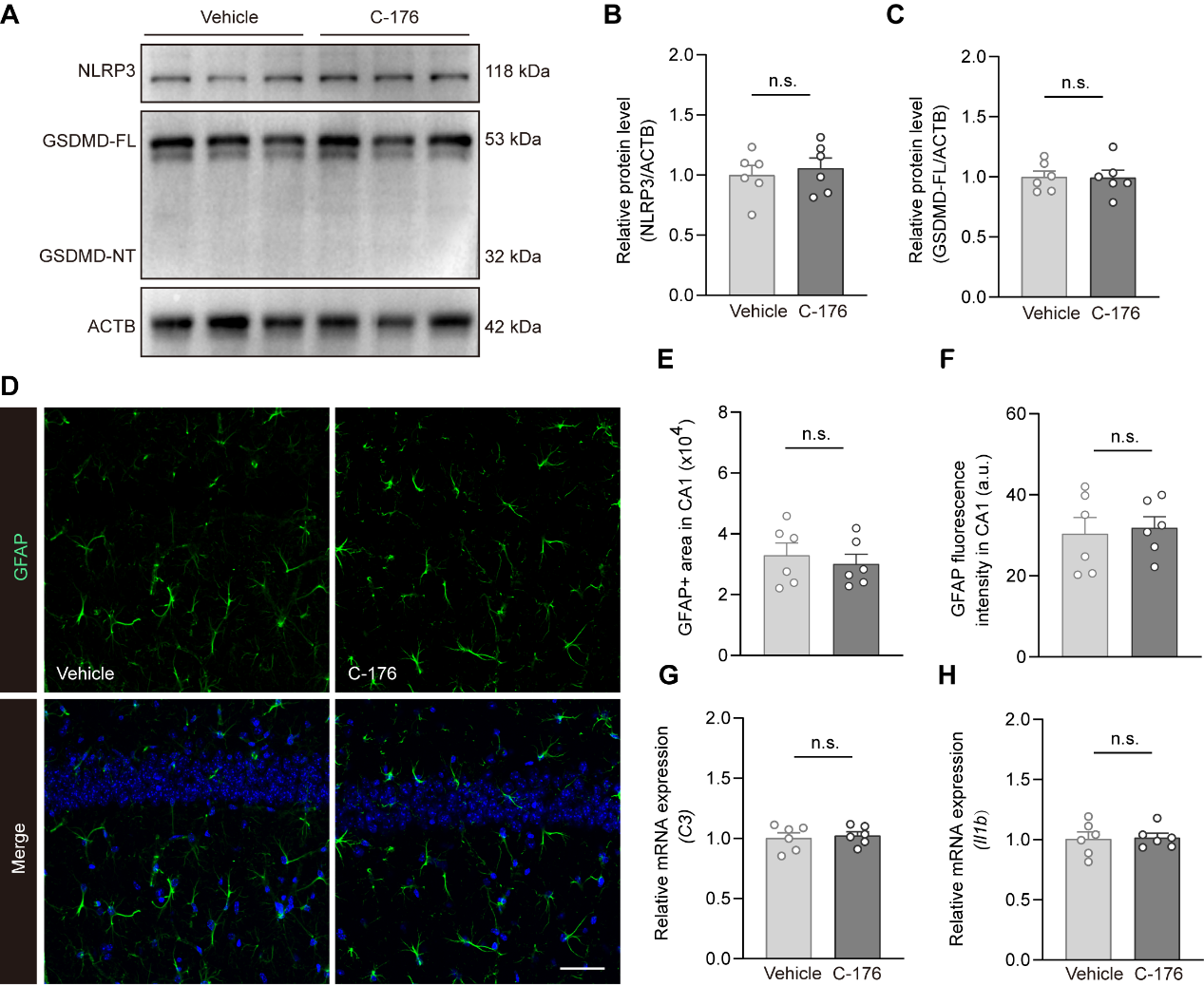


**Figure S14 Inhibition of STING by C-176 does not alter basal pyroptosis or inflammation under physiological conditions.** **(A-C)** Representative immunoblots (A) with statistical analysis of NLRP3 (B) and GSDMD-FL (C) protein levels in mice injected with C-176 or vehicle (n = 6 mice). **(D)** Representative immunofluorescent images of GFAP (green) labeling in the hippocampus (scale bar, 50 μm). **(E-F)** Quantification of GFAP+ area and intensity in the CA1 within the hippocampus (n = 6 mice). **(G-H)** The mRNA expression levels of *C3* (G) and *Il1b* (H) in the hippocampus (n = 6 mice). Data are represented as means ± SEM. Statistical analysis was performed using two-sided unpaired Student’s t-tests. n.s., not significant.
